# Preferential recruitment and stabilization of Myosin II at compartment boundaries in *Drosophila*

**DOI:** 10.1242/jcs.260447

**Published:** 2023-02-24

**Authors:** Jing Wang, Marcus Michel, Lisa Bialas, Giulia Pierini, Christian Dahmann

**Affiliations:** ^1^ School of Science, Technische Universität Dresden, 01062 Dresden, Germany; ^2^ Cluster of Excellence Physics of Life, Technische Universität Dresden, 01062 Dresden, Germany

**Keywords:** *Drosophila*, Myosin II, Compartment boundary, Mechanical tension, Fluorescence recovery after photobleaching, Photoconversion

## Abstract

The regulation of mechanical tension exerted at cell junctions guides cell behavior during tissue formation and homeostasis. Cell junctions along compartment boundaries, which are lineage restrictions separating cells with different fates and functions within tissues, are characterized by increased mechanical tension compared to that of cell junctions in the bulk of the tissue. Mechanical tension depends on the actomyosin cytoskeleton; however, the mechanisms by which mechanical tension is locally increased at cell junctions along compartment boundaries remain elusive. Here, we show that non-muscle Myosin II and F-actin transiently accumulate and mechanical tension is increased at cell junctions along the forming anteroposterior compartment boundary in the *Drosophila melanogaster* pupal abdominal epidermis. Fluorescence recovery after photobleaching experiments showed that Myosin II accumulation correlated with its increased stabilization at these junctions. Moreover, photoconversion experiments indicated that Myosin II is preferentially recruited within cells to junctions along the compartment boundary. Our results indicate that the preferential recruitment and stabilization of Myosin II contribute to the initial build-up of mechanical tension at compartment boundaries.

## Teaser

**Summary:** FRAP and photoconversion experiments reveal that the motor protein Myosin II is preferentially recruited and stabilized at cell junctions during the formation of compartment boundaries in *Drosophila*.

## INTRODUCTION

The generation and modulation of mechanical forces are important for animal development. Mechanical force is involved in shaping cells, tissues and embryos, and also in promoting a tissue-specific cellular organization (Guirao and Bellaïche, 2017; Paci and Mao, 2021; Valet et al., 2021). Mechanical force is often controlled in time and space, i.e. forces are modulated at a particular developmental stage, in a subset of cells within a tissue, or at a particular subcellular localization. For example, during gastrulation of *Drosophila* embryos, a subset of ventral epidermal cells increase their tensile forces at the apicomedial cell cortex, leading to the apical constriction of these cells and subsequent formation of the ventral furrow (Ko and Martin, 2020; Martin et al., 2009). In epithelial cells, tensile forces commonly depend on the activity of non-muscle Myosin II (hereafter Myosin II) motor proteins that lead to the constriction of actomyosin networks associated with the cell cortex (Munjal and Lecuit, 2014). Local increases in tensile forces at specific cell junctions often correlate with local accumulation and activation of Myosin II at the cortex underlying these cell junctions (Bertet et al., 2004; Landsberg et al., 2009; Zallen and Wieschaus, 2004). Several mechanisms have been described that contribute to the accumulation of Myosin II at the cortex of specific cell junctions, including cell–cell signaling (Paré et al., 2019; Paré et al., 2014; Rudolf et al., 2015), localized Rho-GTPase activity (Simoes et al., 2010) and mechanical tension (Fernandez-Gonzalez et al., 2009). However, the cellular mechanisms by which Myosin II accumulates at specific cell junctions remain to be elucidated.

A useful model system to study the mechanisms by which Myosin II is spatially enriched is the sorting of cells within epithelial tissues. During animal development, groups of cells often sort according to their function and identity, contributing to their proper organization into tissues and organs (Fagotto, 2020). A mechanism to separate cells with different identities or function is the local upregulation of actomyosin-dependent tension at boundaries between different cell populations. Classic examples of such boundaries are compartment boundaries, which act as lineage restrictions preventing the intermingling of neighboring groups of cells (known as compartments) (Sharrock and Sanson, 2020; Wang and Dahmann, 2020). Compartments have been identified in numerous vertebrate and invertebrate tissues and organs, including the brain of vertebrate embryos and *Drosophila* adult precursor tissues such as imaginal discs and histoblast nests (reviewed in Dahmann et al., 2011). Cell junctions along compartment boundaries are often characterized by increased levels of Myosin II and by a local increase in mechanical tension (Aliee et al., 2012; Calzolari et al., 2014; Landsberg et al., 2009; Major and Irvine, 2005, 2006; Monier et al., 2010; Umetsu et al., 2014; Urbano et al., 2018). The accumulation of Myosin II at cell junctions along compartments boundaries commonly depends on cell–cell signaling. At compartment boundaries in the embryonic hindbrain of vertebrates, for example, Myosin II accumulation depends on Eph-ephrin signaling (Calzolari et al., 2014; Kindberg et al., 2021). In *Drosophila* larval wing imaginal discs, Myosin II accumulation and increased mechanical tension on cell junctions along the dorsoventral compartment boundary (henceforth DV boundary) depend on Notch signaling (Major and Irvine, 2005; Michel et al., 2016) and along the anteroposterior (AP) compartment boundary (henceforth AP boundary) on Hedgehog signaling (Rudolf et al., 2015). At the AP boundaries in the *Drosophila* embryonic epidermis, it depends on Wingless signaling (Urbano et al., 2018) and on the interaction between the leucin-rich repeat receptor Tartan and the teneurin Ten-m (Paré et al., 2019). However, how these cell–cell signaling pathways result in Myosin II enrichment specifically at cell junctions along these compartment boundaries remains unclear.

**Fig. 1.**
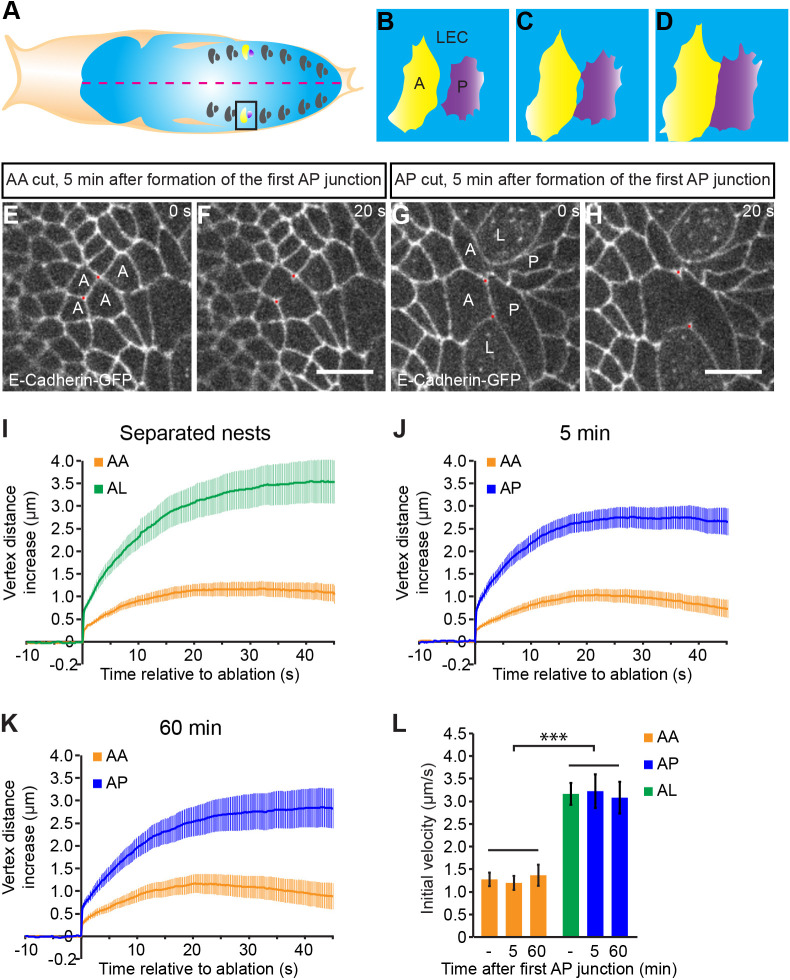
**Mechanical tension is increased during the initiation phase of the AP boundary.** (A–D) Schematic representations of a pupa. Each abdominal segment contains an anterior (‘A’, yellow) and posterior (‘P’, purple) histoblast nest surrounded by larval epidermal cells (LECs, blue) (A). The red dotted line (A) indicates the dorsal midline. During the early pupal stage, A and P histoblast nests are separated by LECs (B). Histoblast nest growth and removal of LECs by extrusion result in progressive contact between cells of the A and P histoblast nests (C), thereby forming the AP boundary (D). (E–H) Representative images from time-lapse movies immediately before and 20 s after laser ablation for control AA cell junctions (E,F) and AP cell junctions (G,H) 5 min after the formation of the AP boundary (defined by the first contact between an A and a P cell). Adherens junctions are labeled by E-cadherin–GFP. Red dots mark the ends of ablated cell junctions. A, anterior cell; P, posterior cell; L, larval epidermal cell (i.e. cells that are initially between A and P cells, but are then removed from the epithelium by extrusion). Scale bars: 10 µm. (I–K) Change in distance between the vertices of cell junctions as a function of time relative to the cut. Junctions between the indicated cell types were cut. Cuts were performed before the formation of the AP boundary (i.e. A and P cells still separated by larval epidermal cells) (I), and 5 min (J) and 60 min (K) after formation of the AP boundary. Mean and s.e.m. are shown (*n*=15 cuts for AA and 15 cuts for AL in six pupae before AP boundary formation; *n*=21 cuts for AA and 16 cuts for AP in eight pupae 5 min after AP boundary formation; *n*=16 cuts for AA and 15 cuts in six pupae for AP 60 min after AP boundary formation). (L) Initial velocity of vertex displacement after ablation of indicated cell junctions and times. Mean and s.e.m. are shown. The initial velocity of vertex displacement after ablation of cell junctions is a relative measure of mechanical tension on the cell junction before the cut. The number of cuts is as in I–K. ***P<0.001 (two-tailed unpaired Student's *t*-test).

The *Drosophila* pupal abdominal epidermis provides a useful system to study how Myosin II is enriched at cell junctions along compartment boundaries. The late pupal abdominal epidermis is subdivided into multiple alternating anterior and posterior compartments (Fig. 1A–D) (Kornberg, 1981). During pupal development, each compartment consists of a small group of anterior or posterior cells, known as histoblast nests (Madhavan and Madhavan, 1980; Ninov et al., 2007; Roseland and Schneiderman, 1979). Individual histoblast nests are initially surrounded, and thus separated from neighboring nests, by larval epidermal cells (LECs). Histoblast proliferation results in the growth of the histoblast nests and the extrusion of intervening LECs. At approximately 18 h after puparium formation, cells of adjacent anterior and posterior histoblast nests make first direct contacts, cell junction by cell junction, thus forming the AP boundaries of the pupal abdominal epidermis. As histoblast nests grow further and more intervening LECs extrude, the AP boundaries extend between the growing adjacent histoblast nests, until all LECs are eventually replaced by the histoblasts. Previous work showed that in late pupal development, when the AP boundaries are fully established, mechanical tension on cell junctions along these AP boundaries is increased compared to mechanical tension on cell junctions in the bulk of the tissue (Umetsu et al., 2014). This local increase in mechanical tension biases cell rearrangements during cell intercalations, thereby contributing to the separation of cells from the anterior and posterior compartments (Umetsu et al., 2014).

Thus far, mainly the mechanisms that maintain cell separation at compartment boundaries have been studied, yet how these boundaries are initially formed remains unclear. It is not known, for example, whether mechanical tension on cell junctions concomitantly increases with the formation of compartment boundaries, and whether and how Myosin II accumulates at cell junctions along the forming compartment boundary. Here, we show that in the *Drosophila melanogaster* pupal epidermis, mechanical tension on cell junctions is increased upon first contact of anterior (A) and posterior (P) cells (i.e. during AP boundary formation), and that Myosin II rapidly, albeit transiently, accumulates at the newly forming AP cell junctions. Moreover, transient Myosin II accumulation correlates with a transient stabilization and recruitment of Myosin II at these junctions. Our data thus reveal mechanisms by which Myosin II is enriched at specific cell junctions during the formation of compartment boundaries.

## RESULTS

### Mechanical tension is increased during the initiation phase of the AP boundary

At a time when anterior and posterior histoblast nests have fused and the AP boundary has been established, mechanical tension is increased by approximately 2.5-fold on cell junctions along the AP boundary compared to cell junctions in the bulk of the histoblasts (Umetsu et al., 2014). To reveal the dynamics of the increase in mechanical tension during the initiation phase of the AP boundary, we probed mechanical tension before the formation of the AP boundary, and 5 and 60 min after the first junction between an anterior and posterior cell had formed. To estimate mechanical tension, we ablated single adherens junctions using focused laser light and recorded using time-lapse imaging the displacement of the two vertices at the ends of the ablated cell junction. We then measured the vertex distance increase and calculated the initial velocity of vertex displacement, which is proportional to the mechanical tension that was present before the junction was ablated (Hutson et al., 2003; Ma et al., 2009). The vertex distance increase and initial velocity of vertex displacement upon ablation of cell junctions between two neighboring anterior histoblasts were comparable for all three time points analyzed (Fig. 1E,F,I–L; Movie 1), indicating that mechanical tension on cell junctions of histoblasts remained constant during the analyzed time period. Upon ablation of cell junctions between anterior histoblasts and LECs, the vertex displacement was elevated compared to that observed upon the ablation of cell junctions between two anterior histoblasts (Fig. 1I; Fig. S1). The initial velocity of displacement increased approximately threefold (Fig. 1L). Interestingly, when we ablated cell junctions along the forming AP boundary (5[Supplementary-material sup1]min after first contact between anterior and posterior histoblasts), the vertex distance increase was elevated and the initial velocity of vertex displacement increased approximately threefold compared to those of cell junctions between two anterior histoblasts (Fig. 1G–J,L; Movie 1). Similarly, at 60 min after the first contact between anterior and posterior histoblasts, the initial velocity of vertex displacement upon ablation of AP cell junctions increased approximately threefold compared to that of cell junctions between two anterior histoblasts (Fig. 1K,L; Fig. S1). These data show that mechanical tension on cell junctions along the AP boundary is increased during the initiation phase of the AP boundary and that the mechanical tension remains increased during the maintenance of the AP boundary.

### Mechanical tension is generated autonomously for each cell

**Fig. 2.**
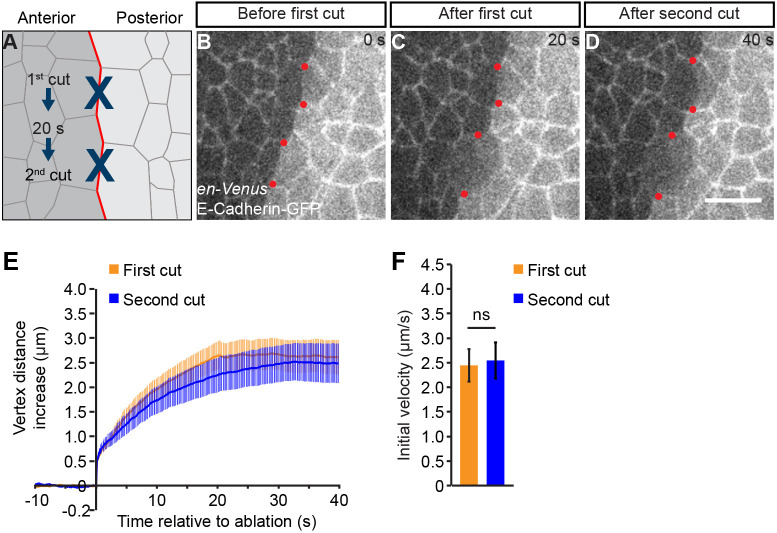
**Mechanical tension is generated autonomously for each cell.** (A) Schematic depicting the experimental strategy. Two adherens junctions along the AP boundary, separated by at least one junction (case 2), were ablated consecutively with a time delay of 20 s. (B–D) Images from a time-lapse movie immediately before the laser cuts and after the first and second laser cuts. Adherens junctions are labeled by E-cadherin–GFP and the posterior compartment is identified by expression of *engrailed-Venus* (*en-Venus*; a marker of the posterior compartment). Red dots mark the ends of ablated cell junctions. Scale bar: 10 µm. (E) Change in the distance between the vertices of the cell junctions of the first and second cuts as a function of time relative to the cut. (F) Initial velocity of vertex displacement after ablation of the indicated cell junctions. For E,F, mean and s.e.m. are shown (*n*=16 first and 16 second cuts in eight pupae). ns, not significant (two-tailed unpaired Student's *t*-test).

The mechanical tension is increased on the first-forming cell junctions at the AP boundary compared to cell junctions in the bulk of the tissue. As the number of cell junctions along the AP boundary increases, collective effects might contribute to the increased tension on cell junctions. For instance, supracellular actomyosin cables, which have been observed along compartment boundaries (Calzolari et al., 2014; Major and Irvine, 2005, 2006; Monier et al., 2010; Paré et al., 2019), have been proposed to enhance junctional mechanical tension (Fernandez-Gonzalez et al., 2009). To test whether the increased mechanical tension along the AP boundary in the abdominal epidermis is a result of collective effects, we used focused laser light to consecutively ablate two neighboring cell junctions along the established AP boundary (Fig. 2A; Fig. S2) (Rudolf et al., 2015). If the mechanical tension of a cell junction along the AP boundary was influenced by forces exerted by the neighboring junctions, then ablating a neighboring junction would result in a decrease in its mechanical tension. We either ablated two immediately neighboring junctions of the same cell (case 1) or two nearby junctions in neighboring cells (case 2) along the AP boundary. In both cases, we ablated the two junctions with a time delay and recorded the resulting displacement of the two vertices at the ends of the ablated cell junctions by time-lapse microscopy. For case 1, the initial velocity of vertex displacement was significantly decreased upon ablation of the second cell junction compared to the initial velocity following ablation of the first cell junction (Fig. S2). However, for case 2, the initial velocity of vertex displacement was indistinguishable upon ablation of the first and second cell junctions (Fig. 2B–F; Fig. S2; Movie 2). Thus, although collective effects might contribute to the mechanical tension of cell junctions within a cell, mechanical tension along the AP boundary is generated autonomously for each cell.

### Myosin II and F-actin transiently accumulate during the initiation phase of the AP boundary

**Fig. 3.**
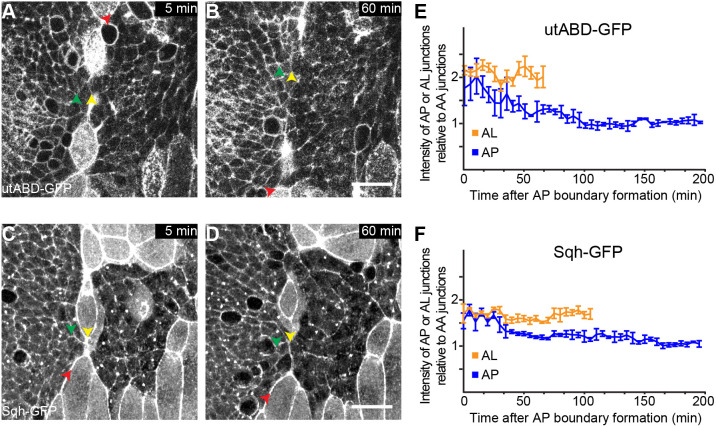
**F-actin and Myosin II are transiently enriched during the initiation phase of the AP boundary.** (A–D) Images from time-lapse movies of histoblasts in the pupal abdomen expressing utABD–GFP to visualize F-actin (A,B) or Sqh–GFP to visualize Myosin II (C,D). Yellow arrowheads mark the positions of the AP boundary, green arrowheads mark adjacent cell junctions in the anterior compartment (AA junctions) and red arrowheads mark junctions between anterior cells and LECs (AL junctions). The time after formation of the AP boundary (i.e. first contact of an anterior and posterior histoblast cell) is indicated. The large cells are LECs. Scale bars: 20 µm. (E,F) Ratio of utABD–GFP (E) and Sqh–GFP (F) intensities between AP junctions or AL junctions and AA junctions as a function of time after AP boundary formation. Mean and s.e.m. are shown (*n*=3 pupae).

Cell junctions along compartment boundaries are often characterized by a local increase of Myosin II and F-actin (Aliee et al., 2012; Calzolari et al., 2014; Landsberg et al., 2009; Major and Irvine, 2005, 2006; Monier et al., 2010; Umetsu et al., 2014; Urbano et al., 2018). To reveal the dynamics of Myosin II and F-actin cortical accumulation, we acquired time-lapse movies to visualize Myosin II [by imaging the regulatory light chain of Myosin II (encoded by *sqh*) fused to GFP (Sqh–GFP)] and F-actin [by imaging the F-actin-binding domain of human utrophin (encoded by *UTRN*) fused to GFP (utABD–GFP)] in the pupal abdominal epidermis at the time of compartment boundary formation (first contact of anterior and posterior histoblast nests). Anterior and posterior nests are initially separated by LECs. LECs extrude one by one and are replaced by the proliferating histoblasts. Cell junctions between anterior histoblasts and LECs had approximately twofold higher levels of utABD–GFP and Sqh–GFP compared to their levels at cell junctions between histoblasts (Fig. 3A,C,E,F). At 5 min after initial contact between an anterior and posterior histoblast, both utABD–GFP and Sqh–GFP levels were elevated approximately twofold at cell junctions at the contact site compared to their levels at cell junctions between histoblasts (Fig. 3A,C,E,F; Movie 3). By 60 min, both utABD–GFP and Sqh–GFP levels at cell junctions between anterior and posterior histoblasts were comparable to their levels at cell junctions in the bulk of the histoblast nests (Fig. 3B,D–F; Movie 3). utABD–GFP and Sqh–GFP levels at cell junctions of LECs remained elevated (Fig. 3E,F). Moreover, Sqh–GFP levels at new cell junctions between histoblasts resulting from the extrusion of an LEC located away from the AP boundary were elevated by approximately 35% compared to Sqh–GFP levels at existing cell junctions (Fig. S3). This finding indicates that the elevated Myosin II levels between A and P cells 5 min after initial contact were not a mere consequence of the LEC extrusion. Finally, immunostaining of the pupal abdominal epidermis using an anti-phospho-Myosin antibody, which reveals active Myosin (Craig et al., 1983; Jordan and Karess, 1997), did not show elevated levels at junctions along the established AP boundary (Fig. S4). We conclude that F-actin and Myosin II transiently accumulate during the initiation phase of the AP boundary.

### Cortical Myosin II localization does not depend on mechanical tension

**Fig. 4.**
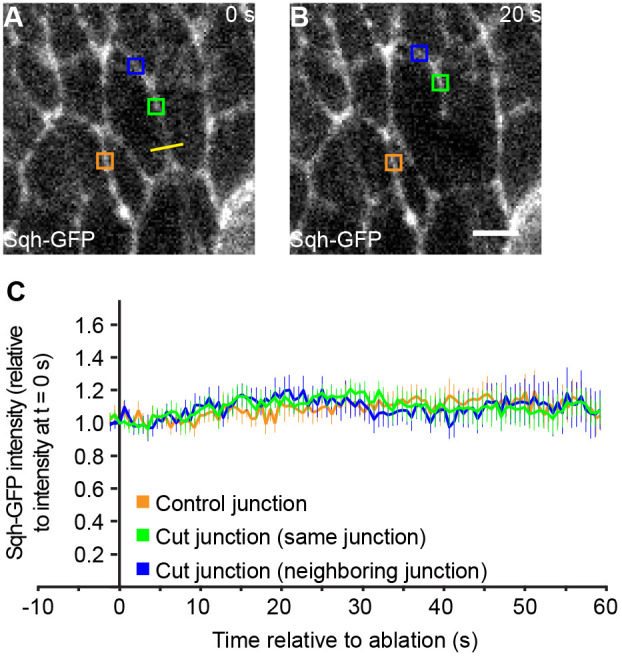
**Cortical Myosin II localization does not depend on mechanical tension.** (A,B) Images from time-lapse movies of histoblasts expressing Sqh–GFP to mark Myosin II at the indicated time points before and after laser ablation of a cell junction. The yellow bar indicates the site of ablation. Colored rectangles show areas in which pixel intensities were measured. Scale bar: 5 µm. (C) Sqh–GFP pixel intensities of the indicated types of cell junctions normalized to the pixel intensity at t=0 s (before the laser cut). The data is plotted as a function of time. The colors of the lines correspond to the colors of the rectangles in A,B. Mean and s.e.m. are shown (*n*=27 junctions of nine pupae).

How does Myosin II accumulate at cell junctions along the AP boundary? Recruitment of Myosin II to the cell cortex can depend on, and be induced by, mechanical tension (Fernandez-Gonzalez et al., 2009; Kobb et al., 2017; Pouille et al., 2009). To test whether Myosin II accumulation on cell junctions along the AP boundary depends on mechanical tension, we ablated a part of a single adherens junction to release the mechanical tension on this junction. We then monitored the level of Myosin II (Sqh–GFP) on the remaining part of the junction or on an immediately neighboring junction by time-lapse microscopy. If cortical localization of Myosin II depended on mechanical tension, we expected that Myosin II levels would decline upon ablation of a junction. At control (unablated) cell junctions, Myosin II levels remained fairly constant over time (Fig. 4A–C; Movie 4). Similarly, upon ablation, Myosin II levels remained fairly constant both on the remaining part of the ablated junction and on immediately neighboring junctions (Fig. 4A–C; Movie 4). These data show that the steady-state levels of cortical Myosin II do not depend on the mechanical tension exerted on that cell junction.

### Myosin II is transiently stabilized during the initiation of the AP boundary

**Fig. 5.**
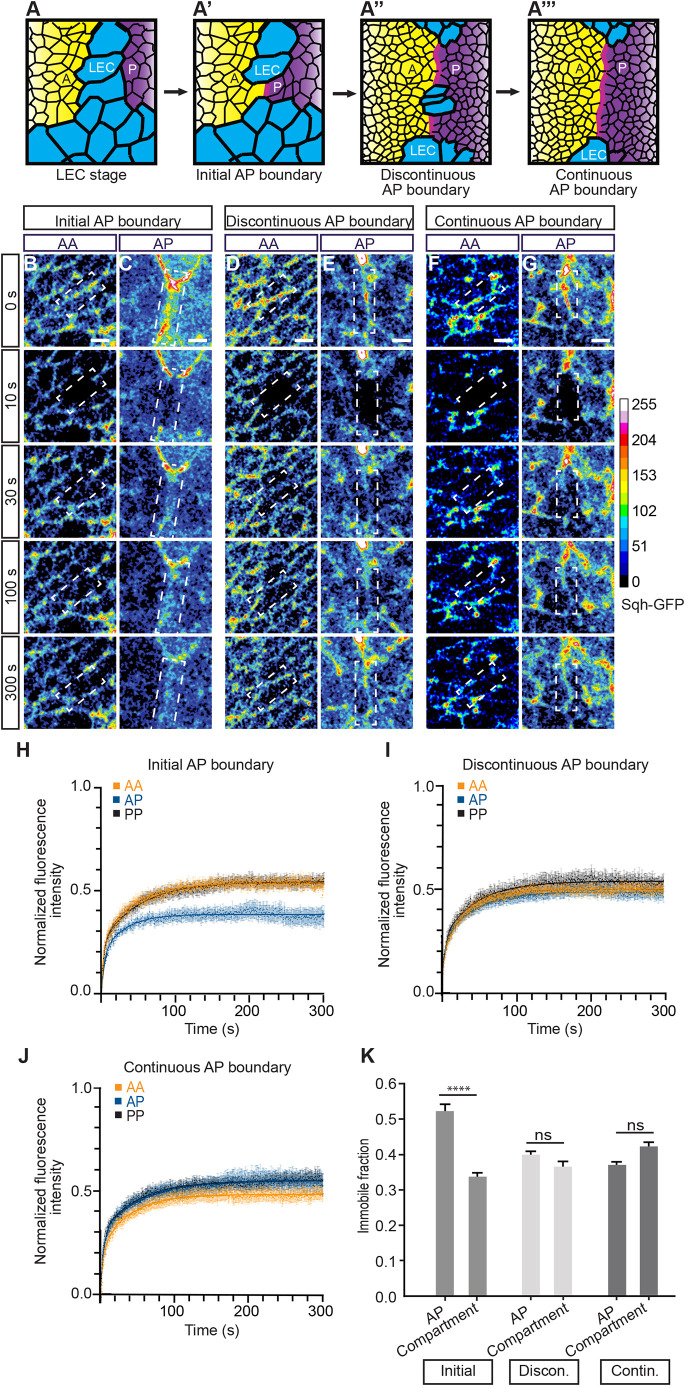
**Myosin II is transiently stabilized during the initiation of the AP boundary.** (A–A‴) Schematics illustrating the developmental stages. (B–G) Representative images from time-lapse movies of FRAP experiments showing histoblasts expressing Sqh–GFP before photobleaching (0 s) and at the indicated times after photobleaching for the indicated developmental phases. The positions of the single photobleached junctions are indicated by white dashed boxes. Colors indicate normalized pixel intensities of Sqh–GFP. Scale bars: 5 µm. (H–J) Normalized fluorescence intensity of Sqh–GFP after photobleaching as a function of time is shown for the indicated developmental phases. The locations of the photobleached junctions are indicated. Mean and s.e.m. are shown. For H, *n*=47 AA, 32 AP and 30 PP junctions analyzed in 20 pupae. For I, *n*=37 AA, 22 AP and 21 PP junctions analyzed in ten pupae. For J, *n*=50 AA, 41 AP and 26 PP junctions analyzed in 18 pupae. The solid lines represent the fitted functions. (K) Immobile fraction of Sqh–GFP is shown for the indicated developmental phases and locations of junctions (compartment: mean of AA and PP). Error bars represent the standard error of the fit. *n* is as in H–J. ns, not significant; ****P<0.0001 (Mann–Whitney test).

Another possible mechanism for Myosin II accumulation on cell junctions along the AP boundary is the stabilization of Myosin II at these junctions. To test this possibility, we performed fluorescence recovery after photobleaching (FRAP) experiments on Myosin II (visualized by imaging Sqh–GFP) and considered the fraction of proteins that did not recover after photobleaching (immobile fraction) as the pool of stable Myosin II at a cell junction. We performed the FRAP experiments at three consecutive developmental phases (Fig. 5A–A‴): the initial AP boundary (formation of the first cell junction between A and P histoblast nests), the discontinuous AP boundary (intervening LECs between histoblasts) and the continuous AP boundary (no intervening LECs). The initial AP boundary phase corresponded to the period during which Myosin II was enriched along the AP boundary (see Fig. 3). For each phase, the Sqh–GFP fluorescence signal was recorded before and after photobleaching of individual cell junctions and the immobile fraction was calculated (see Materials and Methods). The immobile fraction of Sqh–GFP was similar for cell junctions at the AP boundary and within the compartments for the later phases (discontinuous and continuous AP boundary) (Fig. 5D–G,I–K; Movie 5). However, for the initial AP boundary phase, the immobile fraction of Sqh–GFP increased by approximately 52% for cell junctions along the AP boundary compared to its levels at cell junctions within the compartments (Fig. 5B,C,H,K; Movie 5). Thus, Myosin II both accumulates (Fig. 3) and is stabilized at the first forming cell junction of the AP boundary.

### Myosin II is preferentially recruited during the initiation of the AP boundary

**Fig. 6.**
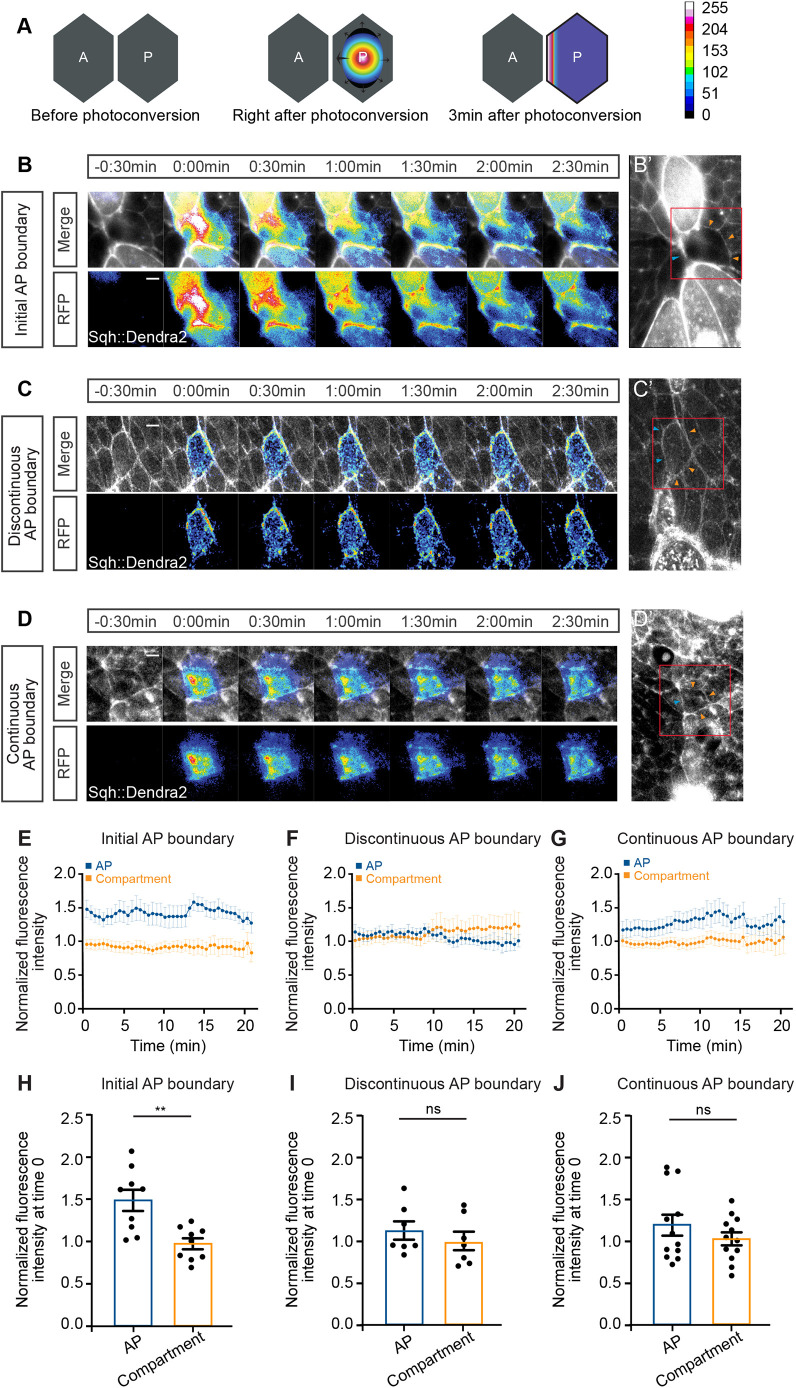
**Myosin II is preferentially recruited during the initiation of the AP boundary.** (A) Schematics illustrating the photoconversion of Sqh::Dendra2 from a green to a red fluorescent protein in a single cell located along the AP boundary. Only the photoconverted protein is depicted. The color scale indicates fluorescence intensities of photoconverted Sqh::Dendra2 in B–D. (B–D) Representative images of time-lapse movies showing the red fluorescence of Sqh::Dendra2 at the indicated times before and after photoconversion for the three developmental phases. Red-channel pixel intensities are shown in color (RFP). Merged images show the overlay of red-channel pixel intensities (color) and the green-channel pixel intensities of non-converted Sqh::Dendra2 (gray). B′–D′ show lower-magnification views before photoconversion. Red boxes in B′–D′ indicate the areas shown in B–D. Blue and orange arrowheads indicate the AP cell junctions and the junctions facing cells of the same compartment, respectively. Scale bars: 5 µm (B–D). (E–G) Red-channel fluorescence intensities of Sqh::Dendra2 at the AP cell junctions and the non-AP cell junctions (compartment) of the same cell as a function of time after photoconversion for the different developmental phases. Fluorescence intensities are normalized to the average fluorescence intensity of the same compartment junctions at the first time point after photoconversion (0 s). (H–J) Normalized fluorescence intensity at t=0 (computed as in E–G) for the different developmental phases. Mean and s.e.m. are shown. For E,H, *n*=8 pupae; for F,I, *n*=7 pupae; and for G,J, *n*=12 pupae. ns, not significant; **P<0.01 (Mann–Whitney test).

Plasma membrane and cortical proteins are recycled through transport from cytoplasmic pools (Iyer et al., 2019). Thus, a further possible mechanism for Myosin II accumulation on cell junctions along the AP boundary is to preferentially recruit Myosin II from the cytoplasm to the cell cortex associated with AP cell junctions. To test this possibility, we expressed a photoconvertible version of Myosin II (Sqh::Dendra2) (Montembault et al., 2017) allowing us to trace a defined pool of Myosin II within cells. Blue light (wavelength of 405 nm) excitation converts Sqh::Dendra2 from a green to a red fluorescent protein. We illuminated the central cytoplasmic region of cells located directly at the AP boundary with blue light at the same developmental phases as before (Fig. 5) and subsequently measured the cortical distribution of the photoconverted Sqh::Dendra2 protein (Fig. 6A; see Materials and Methods). For the later phases (discontinuous and continuous AP boundary), the photoconverted Sqh::Dendra2 protein was uniformly distributed around the cell cortex (Fig. 6C,C′,D,D′,F,G,I,J; Movie 6). However, for the initial AP boundary phase, the photoconverted Sqh::Dendra2 was enriched by approximately 49% on the cell cortex associated with the AP cell junction compared to its localization on the cell cortices associated with junctions facing cells of the same compartment (Fig. 6B,B′,E,H; Movie 6). Thus, Myosin II accumulates (Fig. 3) and is stabilized (Fig. 5) and preferentially recruited at the first-forming cell junction of the AP boundary.

## DISCUSSION

The spatially controlled subcellular accumulation of Myosin II is important for generating local forces and driving tissue morphogenesis. Here, we show that Myosin II transiently accumulates on cell junctions along the forming AP boundary of the *Drosophila* pupal abdomen. Myosin II accumulation is concomitant with an increase in mechanical tension on the junctions at which Myosin II is enriched. Mechanical tension is generated by each cell and does not depend on a supracellular actomyosin cable. Moreover, Myosin II accumulation does not depend on mechanical tension. Rather, both the preferential recruitment of Myosin II from a cytosolic pool to the AP cell junction and the preferential stabilization of Myosin II at these junctions contribute to Myosin II accumulation. Our data suggest mechanisms for Myosin II accumulation at specific cell junctions within a cell.

### The mechanical tension is increased at forming AP cell junctions

Increased mechanical tension is a hallmark of cell junctions along compartment boundaries. Increased tension maintains straight compartment boundaries by correcting perturbations of the compartment boundaries caused by cell division and by biasing junctional remodeling to prevent intercalation between anterior and posterior cells (Monier et al., 2010; Umetsu et al., 2014). Previous work has quantified mechanical tension on cell junctions at a time at which the compartment boundary was already established (Aliee et al., 2012; Landsberg et al., 2009; Michel et al., 2016; Rudolf et al., 2015; Urbano et al., 2018), raising the question of whether changes in the strength of mechanical tension and compartment boundary formation correlate. We have taken advantage of the possibility to live image and manipulate cell junctions during the formation of the AP boundary in the pupal abdominal epidermis. We show that as the first contact between anterior and posterior cells are made (i.e. the AP boundary forms) mechanical tension is increased on these junctions compared to that of non-AP cell junctions. This suggests that mechanical tension on cell junctions along the AP boundary increases rapidly (within minutes), possibly excluding *de novo* protein expression in this process. We note that cells on both sides of the forming AP boundary previously made contact with LECs (extrusion of an LEC between anterior and posterior cells results in the formation of AP cell junctions) and that cell junctions between A cells (and presumably all histoblasts) and LECs are under a similarly high tension compared to AP cell junctions (Fig. 1H). Future work will be required to test whether there is a molecular ‘memory’ of the histoblast-LEC cell junction that contributes to the high tension of the newly forming AP cell junction.

### Mechanical tension is generated autonomously for each cell

Increased mechanical tension on cell junctions can arise by collective effects or cell by cell. During *Drosophila* embryonic germ band extension and wound healing, mechanical tension on aligned cell junctions is higher compared to that on isolated cell junctions, indicating that there is a tissue-level coordination of the actomyosin cytoskeleton (Fernandez-Gonzalez et al., 2009; Kobb et al., 2017). Based on the consecutive ablation of cell junctions of neighboring cells, we previously reported that mechanical tension on cell junctions along the AP boundary in *Drosophila* wing imaginal discs is not influenced by neighboring cell junctions but is rather generated cell by cell (Rudolf et al., 2015). Using a similar approach, we now show that mechanical tension on cell junctions along the AP boundaries of the abdominal epidermis is also generated cell by cell. Recent work by Scarpa et al. (2018), again using a similar approach, instead revealed that mechanical tension on cell junctions along the AP boundary in the *Drosophila* embryonic epidermis depends on an intact actomyosin cable. Thus, compartment boundaries in *Drosophila* appear to differ with respect to the contribution of tissue-level coordination of the actomyosin cytoskeleton towards increased mechanical tension. We note that compartment boundaries in the embryonic epidermis, on the one hand, and wing imaginal disc and abdominal epidermis, on the other hand, also differ in other aspects. The straight shape of compartment boundaries in the embryonic epidermis is maintained by an actomyosin cable-dependent pushing back of dividing cells (Monier et al., 2010). At the AP boundaries of wing imaginal discs and the abdominal epidermis, instead, increased mechanical tension on cell junctions biases cell rearrangements to maintain a straight and sharp interface (Rudolf et al., 2015; Umetsu et al., 2014). Moreover, cell divisions along compartment boundaries within the embryonic epidermis are oriented (Scarpa et al., 2018), whereas this is not the case for cell divisions along compartment boundaries in wing imaginal discs (Landsberg et al., 2009; Major and Irvine, 2005). Future work will be required to reveal whether the differences in respect to compartment boundary shape maintenance and cell-division orientation are related to the absence or presence of a tissue-level coordination.

### Myosin II transiently accumulates along the AP boundary

Cell junctions along compartment boundaries frequently display increased levels of F-actin and Myosin II (Aliee et al., 2012; Calzolari et al., 2014; Landsberg et al., 2009; Major and Irvine, 2005, 2006; Monier et al., 2010; Umetsu et al., 2014; Urbano et al., 2018). We show that at cell junctions along the AP boundaries of the pupal abdominal epidermis, F-actin and Myosin II transiently accumulate, compared to cell junctions elsewhere. An approximal twofold accumulation of F-actin and Myosin II was detectable in a time window of about 30 min after the first contact between an anterior and posterior cell was made, suggesting that the cortical actomyosin network is strengthened in this time window, and that it contributes to the increased mechanical tension. In the subsequent time points at which we quantified Myosin II and F-actin levels, however, both their levels were comparable between the AP boundary and non-AP cell junctions. This latter finding is consistent with a recent report showing that Myosin II accumulation is barely detectable at cell junctions along the established AP boundary (Iijima et al., 2020). Moreover, also at the DV boundary of larval wing imaginal disc, Myosin II only accumulates around the time of DV boundary formation (Aliee et al., 2012; Major and Irvine, 2006), but not during later larval development, although, owing to the lack of live imaging, a precise measurement of the time window during which Myosin II is enriched following DV boundary formation was not provided in these studies. At both the AP boundary of the pupal abdominal epidermis and the DV boundary of larval wing imaginal discs, increased mechanical tension on cell junctions along the established compartment boundary is maintained despite an apparent lack of Myosin II accumulation (Aliee et al., 2012; Umetsu et al., 2014) (this study). How mechanical tension on cell junctions remains increased is not known but may depend on the structure of the cortical actomyosin network, which determines the efficiency by which Myosin II molecules exert forces on cell junctions (Munjal and Lecuit, 2014). Thus, at least for some compartment boundaries, the mechanisms by which mechanical tension is increased on cell junctions along the boundary might differ between the initiation phase of the boundary (actomyosin accumulation) and a later maintenance phase by which the boundary is already established.

### Myosin II accumulation correlates with preferential Myosin II stabilization and recruitment

How does Myosin II accumulate during the initiation phase of the compartment boundary along AP cell junctions? Elegant experiments in the *Drosophila* embryonic epidermis showed that cortical Myosin II accumulation depends on mechanical tension (Fernandez-Gonzalez et al., 2009). Our findings show that Myosin II is maintained on cell junctions even when these junctions are partially ablated and, thus, the mechanical tension is released, indicating that mechanical tension does not cause the recruitment of Myosin II to cell junctions in the *Drosophila* pupal abdominal epidermis. Moreover, using FRAP, we found that the immobile fraction of Myosin II at forming AP cell junctions was increased compared to its levels at cell junctions in the bulk of the tissue. This finding indicates that Myosin II dissociation from the cortical region associated with AP boundary cell junctions is decreased and, hence, Myosin II is stabilized. The developmental phase during which Myosin II is stabilized corresponds to the developmental phase in which Myosin II accumulates at AP cell junctions, suggesting that Myosin II stabilization contributes to its accumulation. Myosin II stabilization has also been previously reported for other junctions on which Myosin II accumulates, for example, at junctions involved in intercalation, internalization and wound healing in the *Drosophila* embryo and in zebrafish embryonic heart formation (Fernandez-Gonzalez et al., 2009; Kobb et al., 2017; Priya et al., 2020; Yu et al., 2021). Myosin II stabilization might therefore be a general mechanism contributing to the accumulation of this motor protein at cell junctions. Myosin II accumulation at cell junctions is influenced by multiple processes, including E-cadherin adhesion (Smutny et al., 2010), actin assembly (Verma et al., 2012), Rho-ROCK and Rap1 signaling (Garcia De Las Bayonas et al., 2019; Smutny et al., 2010), Toll receptor-Src kinase signaling (Paré et al., 2014; Tamada et al., 2021) and G-protein coupled receptor signaling (Dawes-Hoang et al., 2005; Lavalou et al., 2021). Future work will be required to test whether one or more of these processes underlie the stabilization of Myosin II on cell junctions along the AP boundaries in the pupal abdominal epidermis.

Using photoconversion of Sqh::Dendra2 (a photoconvertible form of Myosin II), we show that Myosin II moves from a cytosolic to a cortical pool within cells. Interestingly, Myosin II movement is biased towards the forming AP cell junction, but not during the later developmental phases, indicating that this biased Myosin II movement contributes to the accumulation of Myosin II at AP cell junctions during the initiation of the AP boundary. It is possible that the preferential recruitment of Myosin II towards AP cell junctions could also lead to a faster recovery of Sqh–GFP at these junctions after photobleaching; however, this was not observed (Fig. 5H). We therefore speculate that the cortical pool of Myosin II is much larger than the cytoplasmic pool and that the cortical pool therefore dominates the kinetics of Sqh–GFP recovery after photobleaching. How Myosin II delivery to the cell cortex is biased is currently unknown. Intracellular protein transport is in part mediated by microtubules. Preferentially oriented microtubules have been implicated in the asymmetric distribution of proteins in cells, for example, during the establishment of planar cell polarity in the *Drosophila* pupal wing and adult ovary (Harumoto et al., 2010; Matis et al., 2014; Shimada et al., 2006; Viktorinová and Dahmann, 2013), during segment boundary formation in the *Drosophila* embryo (Bulgakova et al., 2013) and during the delivery of Rho-GEF2 to the cell cortex in *Drosophila* S2 cells (Rogers et al., 2004). It will be interesting to investigate whether microtubules are oriented in cells along the AP boundary and whether microtubules are required for the accumulation of Myosin II at AP boundary cell junctions.

### A two-step model for AP boundary formation

**Fig. 7.**
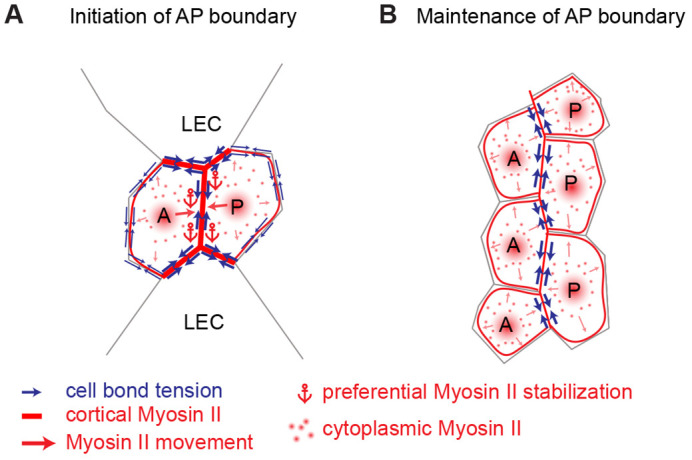
**A two-step model for the formation of the AP boundary.** (A) Removal of LECs by extrusion results in the contact of A and P cells and formation of the AP boundary. AP cell junctions initially accumulate Myosin II. Myosin II accumulation correlates with the delivery of Myosin II from a cytoplasmic pool preferentially to AP cell junctions and the stabilization of cortical Myosin II at these junctions. Mechanical tension on cell junctions along the AP boundary is increased. (B) At a later developmental timepoint, Myosin II is no longer preferentially delivered towards AP cell junctions nor stabilized at the cortex of these junctions. Myosin II is no longer enriched at AP cell junctions. Increased mechanical tension on cell junctions along the AP boundary is maintained.

We propose the following two-step model of how the AP boundary in the pupal abdominal epidermis is formed (Fig. 7). The sequential removal of LECs between anterior and posterior histoblast nests by extrusion results in the contact of A and P cells and, thus, the formation of the AP boundary. Myosin II accumulates at the newly forming AP cell junctions. Myosin II accumulation results from both the directed delivery of Myosin II from a cytoplasmic pool and Myosin II stabilization at the cortex of the AP cell junctions. Myosin II accumulation contributes to the increase in mechanical tension on the newly forming AP cell junction. As more LECs are removed and the AP boundary increases in length, Myosin II is no longer preferentially delivered to AP cell junctions nor preferentially stabilized at these junctions. As a result, Myosin II no longer specifically accumulates on cell junctions along the AP boundary compared to the bulk.

In conclusion, our work shows that Myosin II is preferentially recruited and stabilized to cell junctions along the AP boundary, likely contributing to the increased mechanical tension on these junctions. Asymmetric Myosin II subcellular localization and polarized force generation within cells are hallmarks of epithelia undergoing morphogenesis. It is therefore conceivable that the combination of preferential recruitment and stabilization of Myosin II at cell junctions is a widespread mechanism to spatially regulate mechanical forces during animal development.

## MATERIALS AND METHODS

### Fly stocks and genetics

The following *Drosophila melanogaster* stocks were used: *E-cadherin–GFP* (Huang et al., 2009), *sqh-UTRN::GFP* [expressing the F-actin binding domain of human utrophin fused to GFP (utABD–GFP) (Rauzi et al., 2010)], *sqh^AX3^; sqh-sqh::GFP* [Bloomington *Drosophila* Stock Center (BDSC), #57144], *en-Venus* (Umetsu et al., 2014), *Sqh::Dendra2* (Montembault et al., 2017), *en-Gal4^-54^*, *UAS-DsRed* (BDSC, #6281). The flies were maintained at 25°C on standard fly food.

The genotypes of pupae were as follows: *E-cadherin–GFP*/*E-cadherin–GFP* (Fig. 1; Fig. S1); *en-Venus*, *E-cadherin–GFP*/*E-cadherin–GFP* (Fig. 2); *sqh-UTRN::GFP/sqh-UTRN::GFP* (Fig. 3A,B,E); *sqh^AX3^; sqh-sqh::GFP*/*sqh-sqh::GFP* (Fig. 3C,D,F; Fig. S3); *sqh^AX3^; sqh-sqh::GFP*/*sqh-sqh::GFP* (Fig. 4); *sqh^AX3^; sqh-sqh::GFP*/*sqh-sqh::GFP* (Fig. 5); *Sqh::Dendra2*/*TM6B* (Fig. 6); and *en-Gal4*, *UAS-DsRed*, *E-cadherin–GFP*/*E-cadherin–GFP* (Figs S2 and S4).

### Immunohistochemistry

Staged pupae were dissected and stained as described before (Wang and Yoder, 2011). Briefly, pupae were cut into halves with a scalpel, the interior washed out and the remaining fixed with 4% formaldehyde. Samples were stained with a rabbit anti-phosphorylated-Myosin-regulatory-light-chain antibody (Cell Signaling Technology, 3671, 1:50). The secondary antibody was goat anti-rabbit Cy5 (Jackson ImmunoResearch, 111-175-144, 1:200).

### Time-lapse imaging

Flies were raised under standard conditions. To stage the pupae, white pupae (0 h after puparium formation) were collected and raised at 25°C. The late pupae were placed on a piece of double-sided tape, a window in the pupal case was opened and covered with halocarbon oil 700 (Sigma-Aldrich, H8898). The prepared pupae were then mounted on a glass bottom dish (MatTek Corporation) with the opening facing the cover slip (Ninov and Martín-Blanco, 2007). Imaging was performed using an inverted confocal laser-scanning microscope (Leica SP5 or Zeiss LSM 880) with 40× water-immersion objectives. Image stacks of abdominal segments 2–4 were acquired using 5-min intervals. The *z*-distance between slices in the image stacks was 1 µm.

### Image processing and analysis

For Fig. 3, maximum-intensity projections of the slices containing the fluorescence signal (two slices for F-actin, eight to nine slices for Myosin II) were performed using Fiji (Schindelin et al., 2012). In the resulting images, the cell junctions at the AP boundary or the control border (located one cell row away from the AP boundary) were marked using the line tool (line width 3 pixels) and average pixel intensity was quantified.

For Fig. S3, sum intensity projections of the slices containing the fluorescence signal (two to three slices) were performed using Fiji (Schindelin et al., 2012). The intensity was measured using the line tool (line width 2 pixels) in Fiji and average pixel intensity was measured for the projections in Fig. S3 and on single slices in Fig. S4.

### Laser ablation

Laser ablation was performed as described previously (Umetsu et al., 2014). Pupae of the appropriate genotype were staged and prepared as for the time-lapse imaging. For Figs 1, 2 and 4, and Fig. S1, pupae were imaged on an inverted spinning-disc microscope (Zeiss Axio Observer.Z1 with a Yokogawa CSU10 head and an Andor iXon DU-897 camera) with a 63×/1.2 water-immersion objective. Images were recorded every 0.2 s over a total time of 1 min. Ablation was performed using a pulsed, third harmonic solid-state ultraviolet laser (355 nm, 400 ps, 20 mJ/pulse) focused on the level of the adherens junctions. Tissue relaxation was analyzed manually using Fiji (Schindelin et al., 2012) and plotted as relative distance increase over time or as initial velocity. The initial velocity was calculated by measuring the vertex distance increase between the time points before ablation and the first time point after ablation and dividing it by the time interval (0.2 s).

For Fig. S2, pupae were imaged on an inverted Leica SP8 MP laser scanning microscope with a 63×/1.3 glycerol-immersion objective. Images were recorded every 0.33 s. Ablation was performed using an InSight X3 infrared laser at 800 nm. The initial velocity was calculated as described above. For Fig. 1, time-lapse imaging was started at a stage during which anterior and posterior histoblast nests were still separated, thus allowing the identification of the position and timing of AP boundary formation. For Fig. 4, average Sqh–GFP pixel intensities were measured in an 8×8 pixel mask overlying the adherens junction.

### FRAP

Pupae were staged and prepared as for time-lapse imaging. FRAP was performed on an Olympus IX81 microscope equipped with a 60×/1.35 oil-immersion objective lens (U Plan SApo) and a Yokogawa CSU-X1 spinning-disc scan head. The AP boundary was identified by the shape of the anterior and posterior histoblast nests and the position of extruding LECs. Multiple single cell junctions along the AP boundary and within the histoblast nests (located far apart from each other) were identified in a single field of view and cell junctions were photobleached using a 488 nm laser at 70% power with a dwell time of 400 µs and 20 repetitions. Single-image planes were acquired every 0.5 s. A total of 20 pre-bleached and 600 post-bleached frames per cell junction were acquired.

To obtain recovery curves from the acquired images, we performed the following analysis in MATLAB (www.mathworks.com). At first, the regions corresponding to the background (*BG*), junctions of interest (*J*, including their two vertices) and reference tissue (*REF*, bulk of histoblast nests) were selected manually and their average values (*a*) were computed for each time [*a*_*BG*_(*t*), *a*_*J*_(*t*) and *a*_*REF*_(*t*)].

The average values were then corrected for the background:

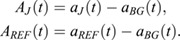
The obtained corrected intensities *A* were then normalized to the prebleach intensity *A*_*pre*_ and the ratio between the junction intensity and the reference was computed to account for overall intensity fluctuations and photobleaching of the tissue:

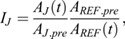
where

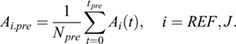


The recovery curves were obtained by setting the prebleach levels to 1 and the levels immediately after photobleaching to 0.

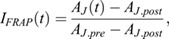
where

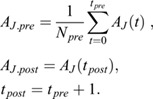
The recovery curves were then averaged over the same type of photobleached junctions (in the anterior or posterior compartment or at the AP boundary).

In order to extract the amount of recovered Myosin II, a double exponential function was fitted to the data:

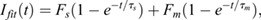


where *F*_*s*_, *τ*_*s*_ and *F*_*m*_, *τ*_*m*_ represent the mobile fraction and the characteristic time in second scale and minute scale, respectively. The fit was performed with the *fitlnm* function in MATLAB and weighted over the standard deviation.

Finally, the immobile fraction was estimated as:

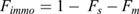
and its error computed from the standard error of the fit parameters (Δ*F*_*s*_, Δ*F*_*m*_) with the error propagation equation:

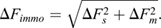


### Photoconversion

Pupae were staged and prepared as for time-lapse imaging, except that pupae were maintained in the dark and the preparation was performed under red light to avoid precocious photoconversion. Photoconversion was performed on a Zeiss LSM 980 Airyscan 2 microscope with a 63×/1.35 oil-immersion objective. Single cells located directly along the AP boundary were identified as described for the FRAP experiment. Sqh::Dendra2 was photoconverted in the cytosolic region of these single cells by exposing this region to 405 nm laser light at 2% power with 100 iterations. For efficient photoconversion, approximately 80% of the cytosolic region was exposed to the laser light, carefully avoiding exposing the cell cortex. Image stacks covering five image planes, each 1 µm apart, were acquired every 30 s using a 488 nm laser at 2.5% power and a 561 nm laser at 3% power. A total of five pre-bleached and 30–40 post-bleached frames per cell were acquired.

To test whether Myosin II was enriched at the AP boundary, we compared the intensity of the photoconverted protein at the junction along the AP boundary with the junctions facing cells of the same compartment. First, we identified the background (*BG*) by measuring the pixel intensities of ten distinct regions for each pre-bleached frame using Fiji (Schindelin et al., 2012). Second, we identified the junctions of interest [one at the AP boundary (*AP*) and three facing the same compartment (*C_i=_*_1,2,3_)] and manually selected regions covering these junctions. We averaged the fluorescence intensities in each region [*a*_*BG*_(*t*), *a*_*AP*_(*t*) and 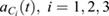] and subtracted the background levels from the regions of interest:

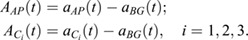
We then normalized the intensities at the AP boundary junction to the one at the same compartment junctions at the first time point after photoconversion (*t=0 s*); an analogous normalization was applied to the same compartment junctions as follows:

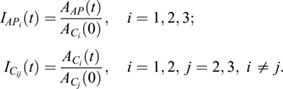
The overall normalized intensity for the AP boundary junction and same compartment junctions were computed by averaging over the three same compartment junctions:

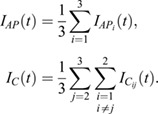
The same pipeline was applied to all three different developmental stages.

### Statistical analysis

Statistical significance was tested in Figs 1 and 2 using a two-sample, two-tailed unpaired Student's *t*-test; in Fig. S4 using a two-sample, two-tailed paired Student's *t*-test; in Fig. S2 using a one-sample Wilcoxon signed-rank test within the R package (R Foundation for Statistical Computing; https://www.R-project.org/); and in Figs 5 and 6 using a two-sample, unpaired Mann–Whitney test.

## Supplementary Material

10.1242/joces.260447_sup1 Supplementary information
