# Supplementary material for: Preferential recruitment and stabilization of Myosin II at compartment boundaries in Drosophila
Source: J Cell Sci. 2023 Feb 24;136(5):jcs260447. doi: 10.1242/jcs.260447 (PMC10022687; doi:10.1242/jcs.260447)
Supplement: Supplementary information [file joces-136-260447-s1.pdf]

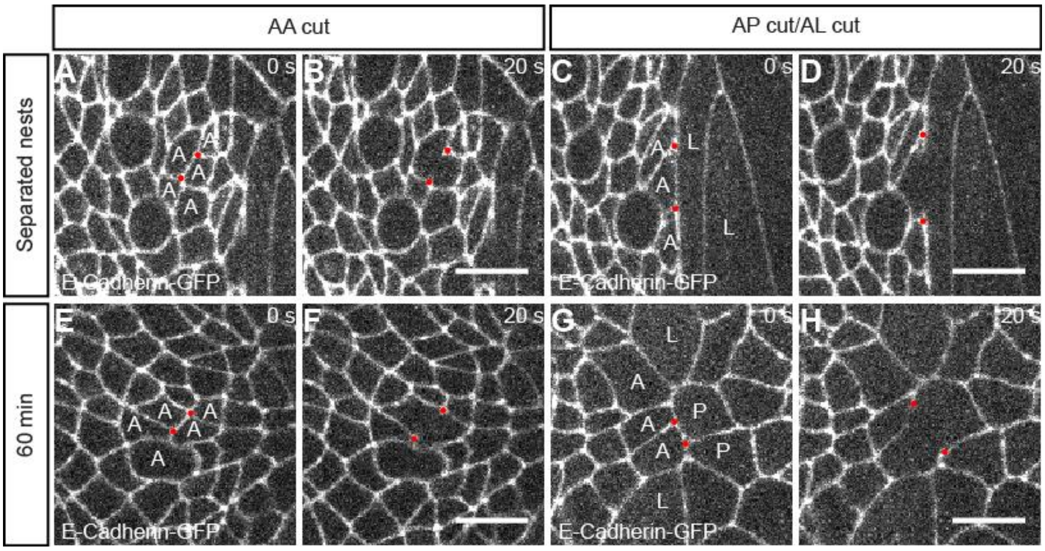

Fig. S1. Mechanical tension remains elevated at the established AP boundary

(A-H) Images from time-lapse movies immediately before and 20 sec. after laser cut for (A,B,E,F) AA cell junctions and (C,D,G,H) AL cell junctions before (separated nests) and 60 min. after formation of the AP boundary (as defined by the first contact between an A and a P cell). Adherens junctions are labeled by E-cadherin-GFP. Red dots mark ends of ablated cell junctions. A: anterior cell; P: posterior cell; L: larval epidermal cell (i.e. cells that are initially between A and P cells, but are then removed from the epithelium by extrusion). Scale bar is 10 µm.

Journal of Cell Science • Supplementary information

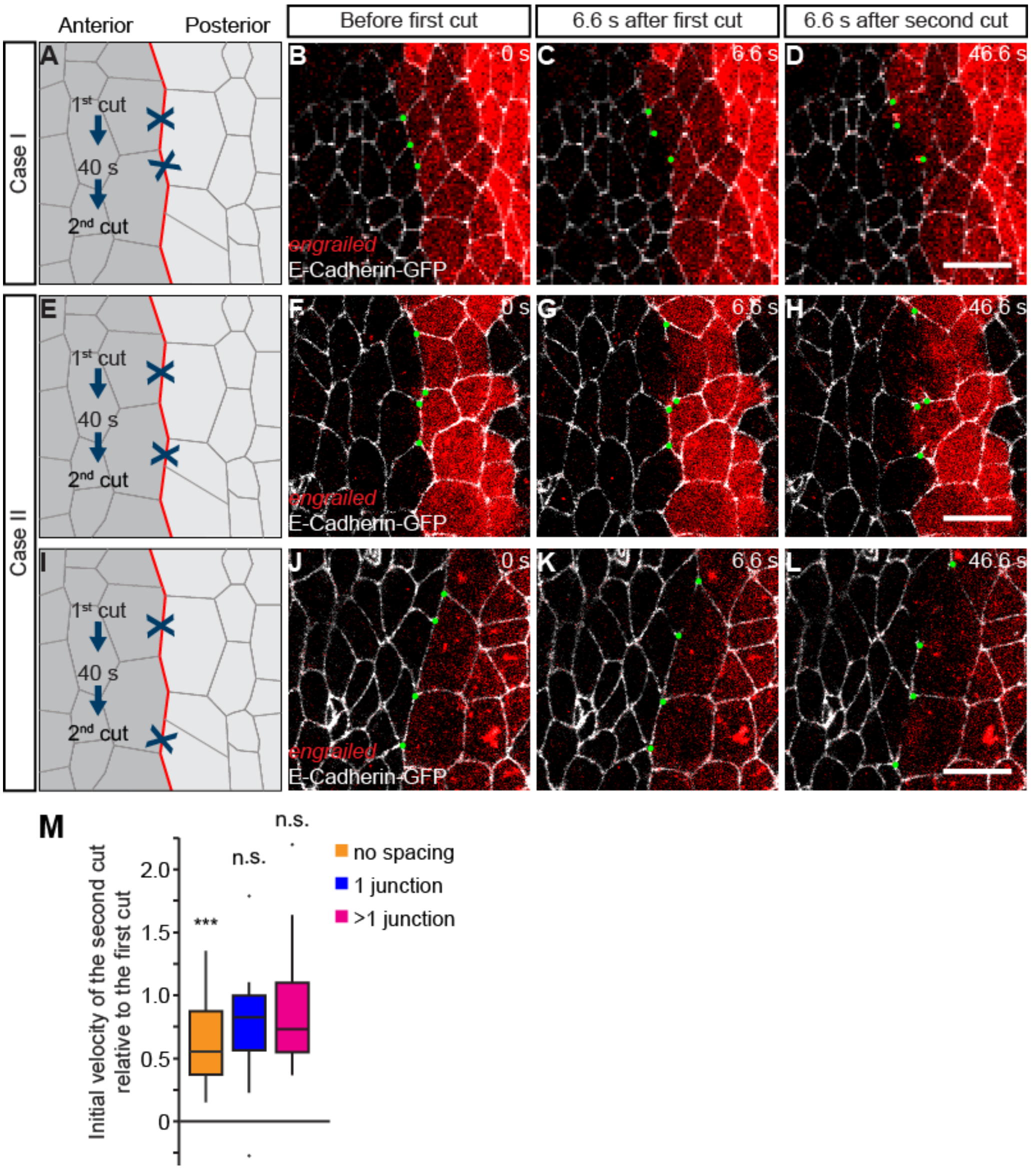

Fig. S2. Mechanical tension is generated autonomously for each cell

(A) Schematic drawing depicting the experimental strategy. Two immediately neighboring adherens junctions along the AP boundary in the same cell (case I, here a posterior cell) are ablated consecutively with a time delay of 40 s.

(B-D) Images from a time-lapse movie immediately before laser cut and after first and second laser cuts (times in seconds) for two immediately neigboring adherens junctions.

(E) Schematic drawing depicting the experimental strategy. Two adherens junctions along the AP boundary separated by one junctions (case II, different cells) are ablated consecutively with a time delay of 40 s.

(F-H) Images from a time-lapse movie immediately before laser cut and after first and second laser cuts (times in seconds) for two adherens junctions separated by one junction.

Journal of Cell Science • Supplementary information

(I) Schematic drawing depicting the experimental strategy. Two adherens junctions along the AP boundary separated by two junctions (case II, different cells) are ablated consecutively with a time delay of 40 s.

(J-L) Images from a time-lapse movie immediately before laser cut and after first and second laser cuts (times in seconds) for two adherens junctions separated by two junctions.

In (B-D, F-H, J-L) adherens junctions are labeled by E-cadherin-GFP and the posterior compartment is identified by expression of *engrailed-Gal4 UAS-DsRed* (a marker of the posterior compartment). Red dots mark ends of ablated cell junctions. Scale bars are 10 µm.

(M) Ratio of initial velocities of vertex displacement after first and second cut for immediately neighboring junctions (no spacing) or for junctions separated by one or more than one junction. Mean and SEM are shown (n= 16 cuts of 14 pupae for no spacing; n= 14 cuts of 14 pupae for 1 junction spacing; n= 18 cuts of 7 pupae for >1 junction spacing. ***: $p<0.001$, n.s.: not significant. Wilcoxon signed-rank test.

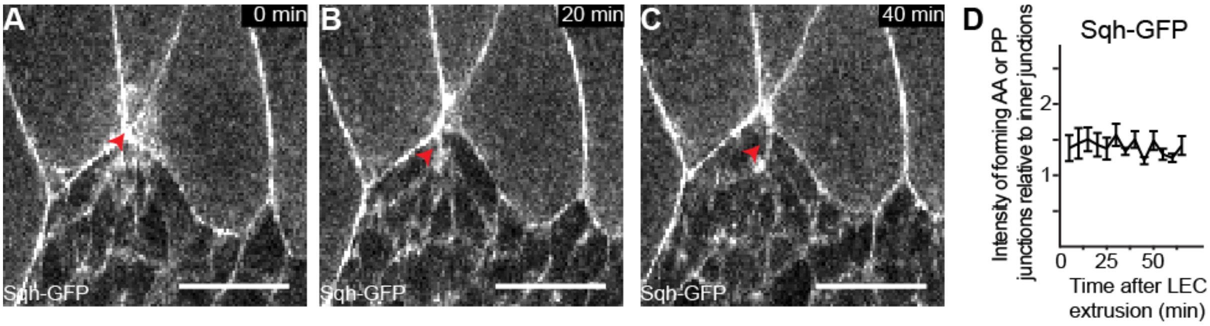

Fig. S3. Myosin II is only slightly increased at newly forming junctions between histoblasts located away from the AP boundary

(A-C) Images from time-lapse movies of histoblasts in the pupal abdomen expressing Sqh-GFP to visualize Myosin II. The arrowhead in (A) marks the position of the extruded larval epidermal cell (LECs) which is away from the AP boundary. The arrowheads in (B,C) mark the new junction formed between two histoblasts. Time after extrusion of the LEC marked in (A) is indicated. The large cells are remaining LECs. Scale bars are 20 µm.

(D) Ratio of Sqh-GFP intensities of histoblast junctions newly formed after LEC extrusion away from the AP boundary and existing histoblast junctions elsewhere (but not in contact with LECs) as a function of time after LEC extrusion. Mean and SEM are shown (n = 4 pupae).

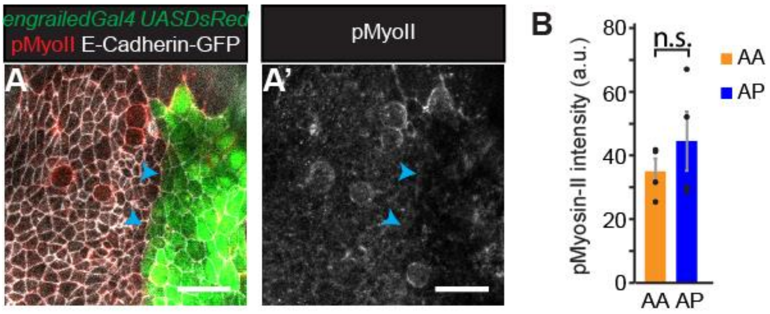

Fig. S4. Phospho-Myosin is not elevated at the established AP boundary

(A) Image of the abdominal epidermis of a pupa 18-20 h APF expressing E-cadherin-GFP (grey, to mark adherens junctions) and DsRed under the control of *engrailed* (*engrailed-Gal4, UAS-DsRed*, green, to mark the posterior compartment) stained for phospho-Myosin. Blue arrowheads point to exemplary AP junctions.

(B) Phospho-Myosin intensity at AP and AA adherens junctions. Mean and SEM are shown (n = 4 pupae). n.s.: not significant. Paired Student's *t*-test.

Journal of Cell Science • Supplementary information

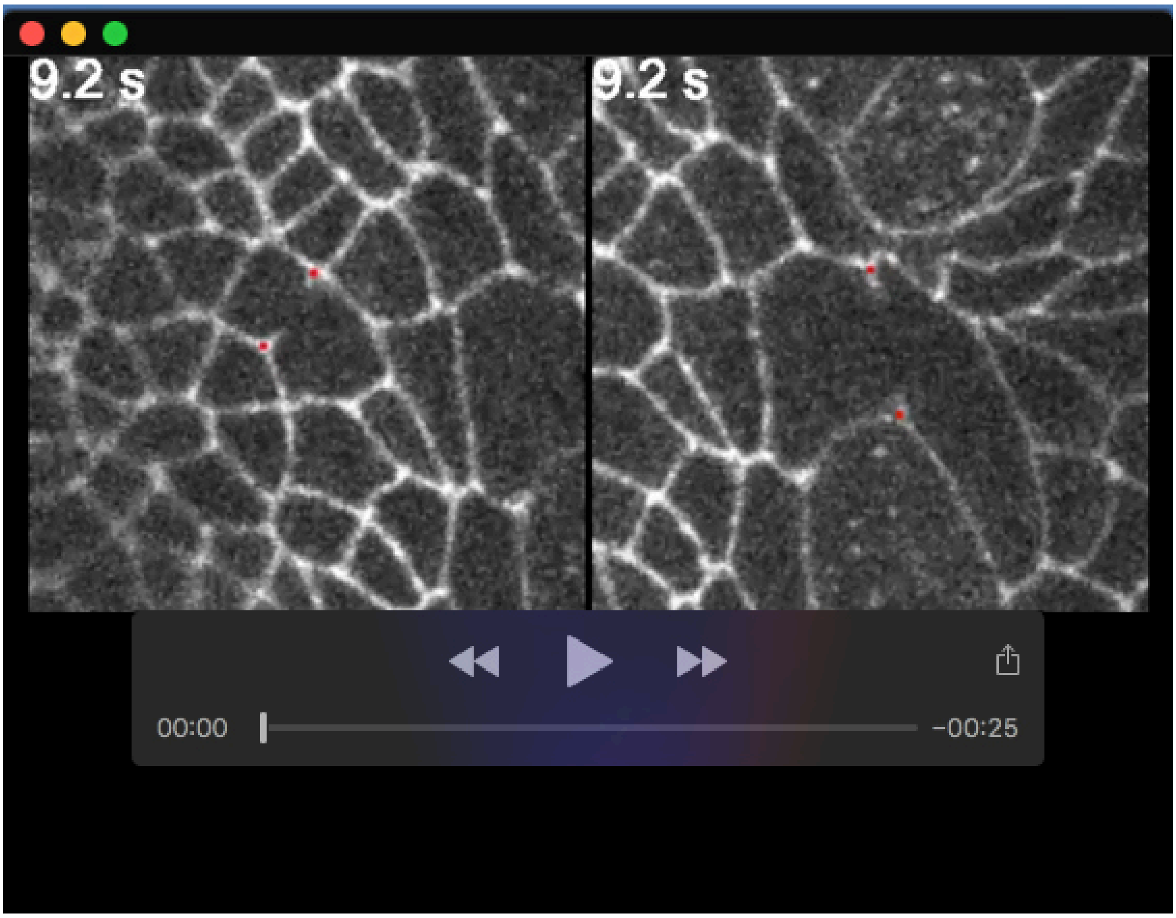

Movie 1. Mechanical tension is rapidly increased during the initiation phase of the AP boundary

Time-lapse movies showing dorsal views of an abdominal epidermal segment of a pupa 5 min. after formation of the AP boundary (as defined by the first contact between an A and a P cell). Adherens junctions, labelled by E-cadherin-GFP, are depicted before and after ablation of a control AA cell junction or an AP cell junction. Times relative to the laser cut are indicated. Red dots indicate the two ends of the ablated cell junction. Scale bars: 10 µm.

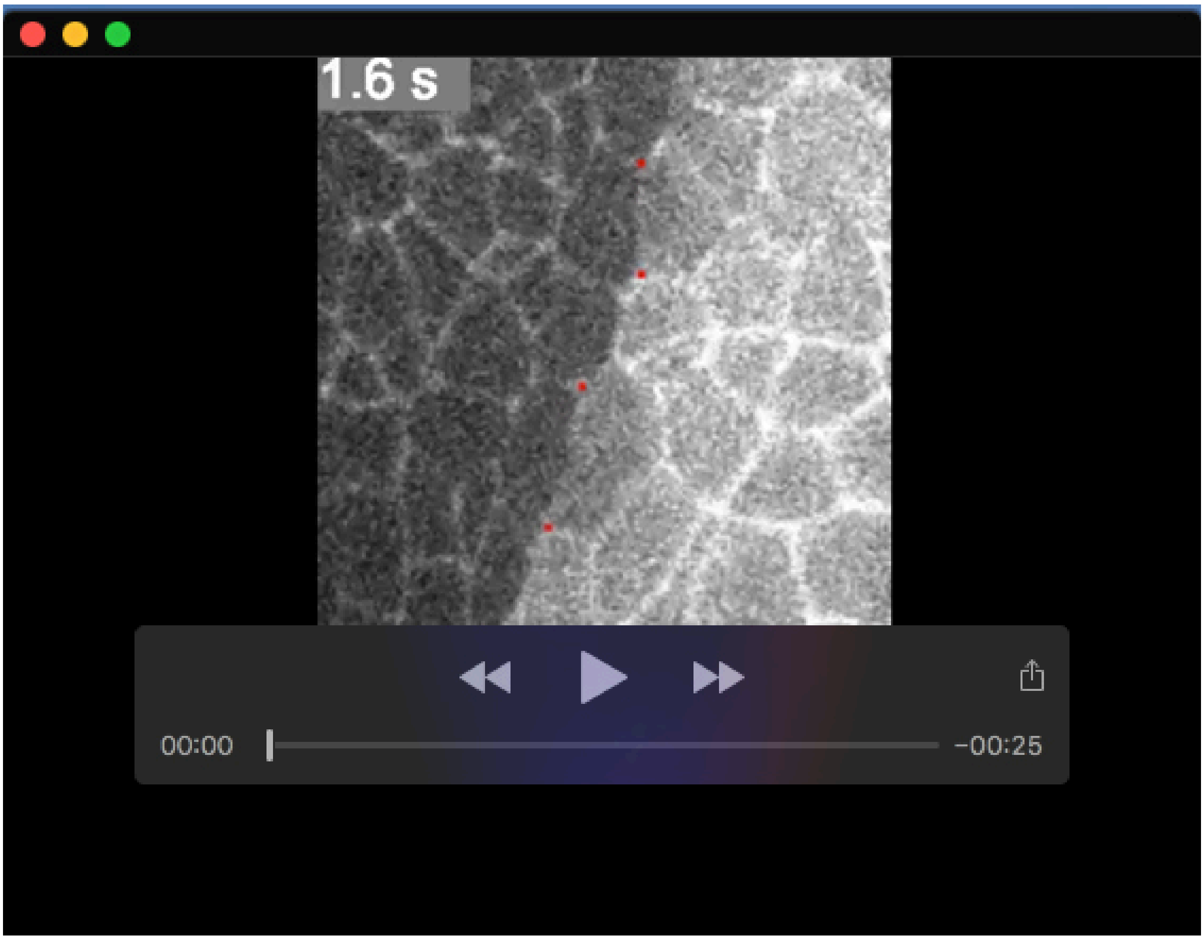

Movie 2. Mechanical tension is generated autonomously for each cell

Time-lapse movie of histoblasts in the pupal abdominal epidermis immediately before laser cut and after first and second laser cuts. Times relative to the first laser cut are shown. Adherens junctions are labelled by E-cadherin-GFP and the posterior compartment is identified by expression of *engrailed-Venus* (a marker of the posterior compartment). Red dots mark ends of ablated cell junctions. Scale bar is 10 μm.

Journal of Cell Science • Supplementary information

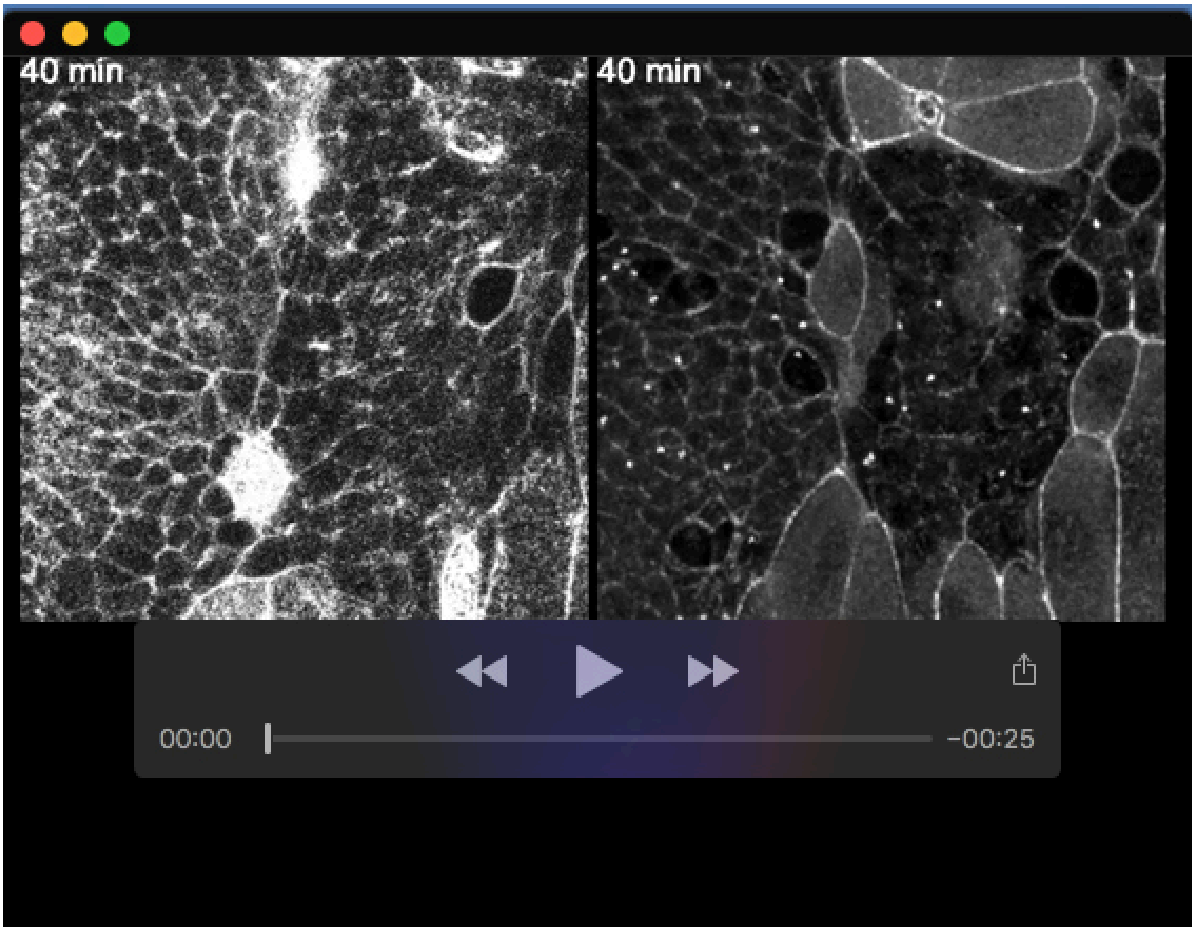

Movie 3. F-actin and Myosin II are transiently enriched during the initiation phase of the AP boundary

Time-lapse movies of histoblasts in the pupal abdominal epidermis expressing utABD-GFP to visualize F-actin (left) or Sqh-GFP to visualize Myosin II (right). Times relative to the first contact between A and P cells are indicated. Large cells are larval epidermal cells (LECs). LECs extruding at the future site of the AP boundary are labeled by an ‘L’. Scale bars are 20 µm.

Journal of Cell Science • Supplementary information

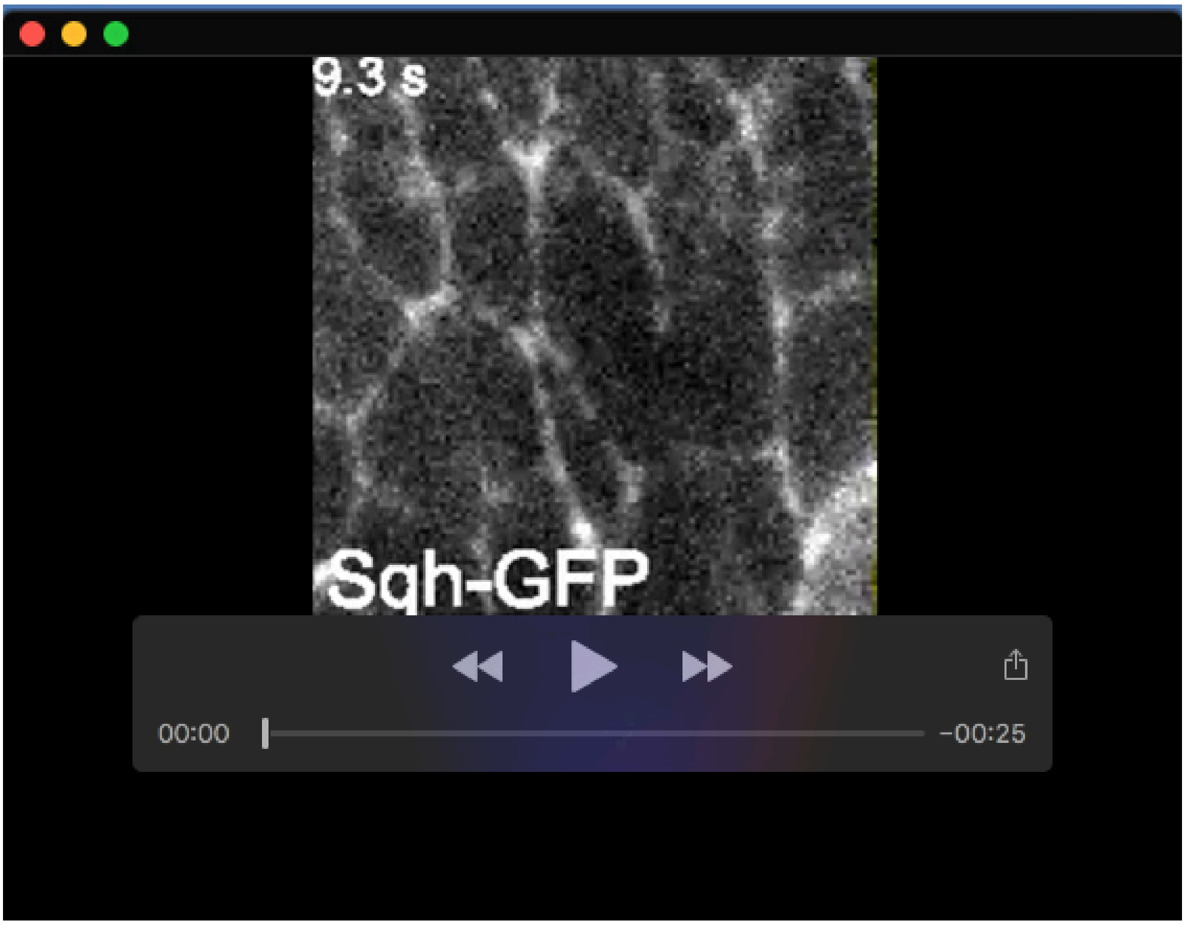

Movie 4. Cortical Myosin II localization does not depend on mechanical tension

Time-lapse movie of cell junctions of histoblasts expressing Sqh-GFP to mark Myosin II. A single cell junction is cut by laser light. Time relative to the laser cut is indicated. Scale bar is 5 µm.

Journal of Cell Science • Supplementary information

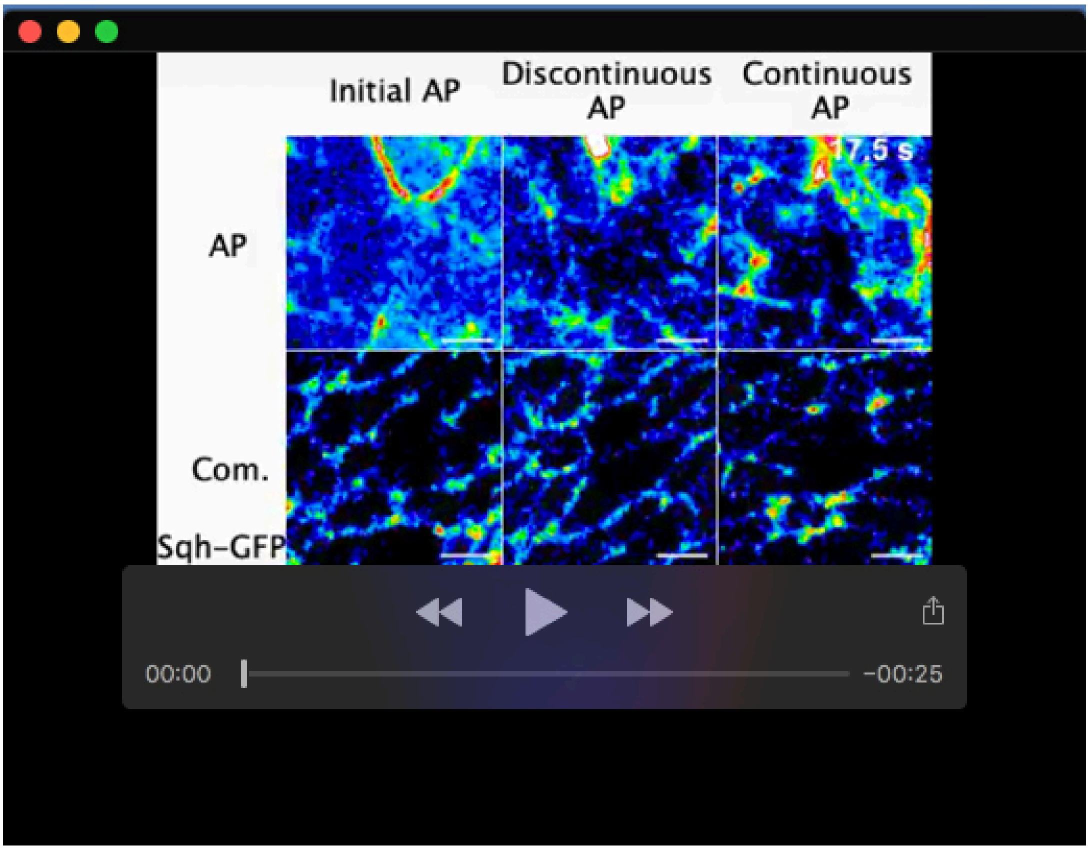

Movie 5. Myosin II is transiently stabilized during the initiation of the AP boundary
Time-lapse movies of FRAP experiments showing histoblasts expressing Sqh-GFP at the three developmental phases. Sqh-GFP was photobleached at AP cell junctions or at cell junctions within a compartment, as indicated. Time relative to photobleaching is shown. Colors indicate pixel intensities of Sqh-GFP (see Fig. 5B-G). Scale bars are 5 µm.

Journal of Cell Science • Supplementary information

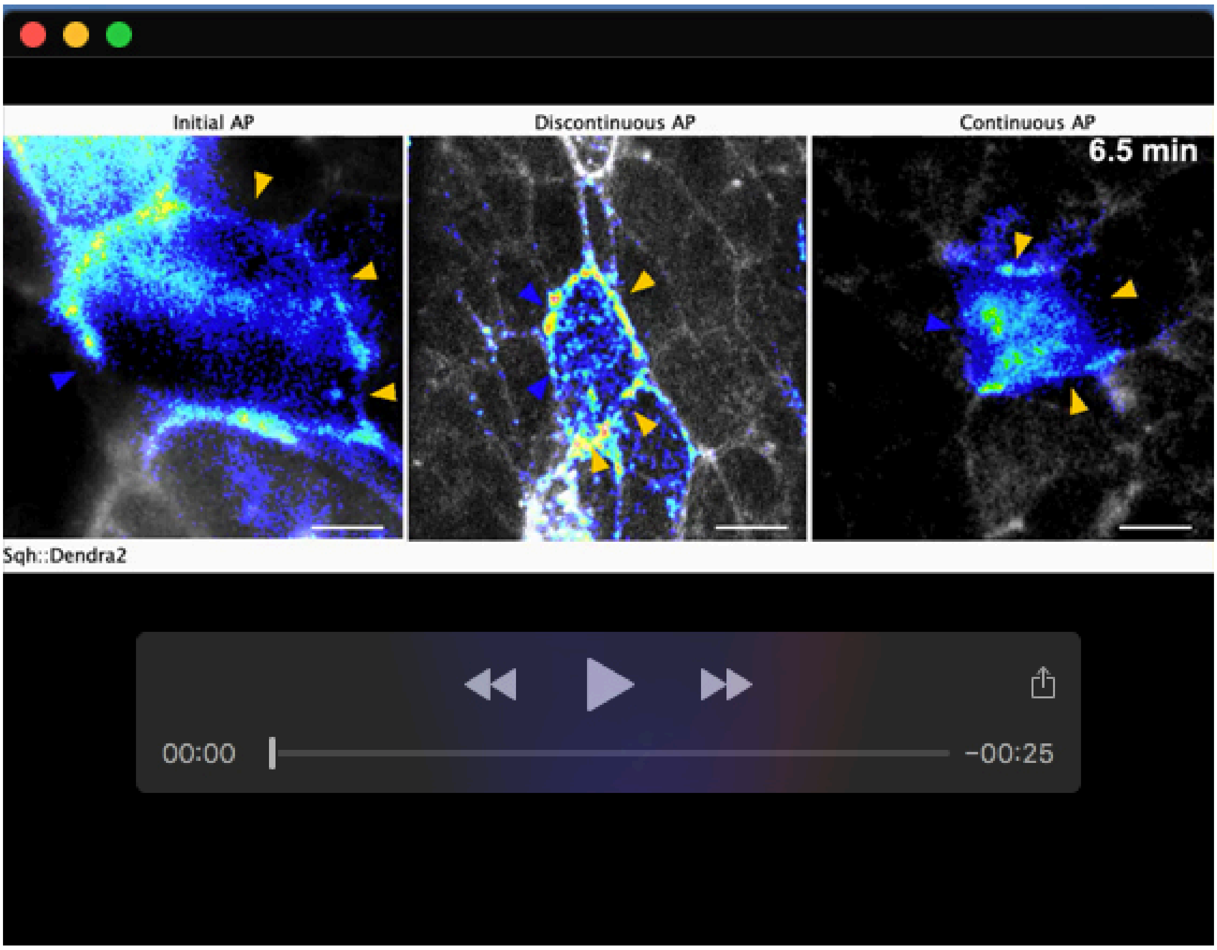

Movie 6. Myosin II is preferentially recruited during the initiation of the AP boundary
Time-lapse movies showing the photoconversion of Sqh::Dendra2 at the three developmental phases. A merge overlaying red channel pixel intensities of photoconverted Sqh::Dendra2 (color) and the green channel pixel intensities of non-photoconverted Sqh::Dendra2 (grey) is shown. Blue and orange arrowheads point towards the AP cell junctions and the junctions facing cells of the same compartment, respectively. Scale bars are 5 µm.
