# Supplementary material for: Preferential recruitment and stabilization of Myosin II at compartment boundaries in Drosophila
Source: J Cell Sci. 2023 Feb 24;136(5):jcs260447. doi: 10.1242/jcs.260447 (PMC10022687; doi:10.1242/jcs.260447)
Supplement: Supplementary file 1 [file joces-136-260447_review_history.pdf]

# Preferential recruitment and stabilization of Myosin II at compartment boundaries in *Drosophila*

Jing Wang, Marcus Michel, Lisa Bialas, Giulia Pierini and Christian Dahmann
DOI: 10.1242/jcs.260447

**Editor**: Anne Straube

**Review timeline**
Original submission: 21 July 2022
Editorial decision: 1 September 2022
First revision received: 8 January 2023
Accepted: 23 January 2023

**Original submission**

First decision letter

MS ID#: JOCES/2022/260447

MS TITLE: Preferential recruitment and stabilization of Myosin II at compartment boundaries in Drosophila

AUTHORS: Jing Wang, Marcus Michel, Lisa Bialas, Giulia Pierini, and Christian Dahmann
ARTICLE TYPE: Research Article

We have now reached a decision on the above manuscript.

To see the reviewers' reports and a copy of this decision letter, please go to: https://submit-jcs.biologists.org and click on the 'Manuscripts with Decisions' queue in the Author Area. (Corresponding author only has access to reviews.)

As you will see, the reviewers raise a number of substantial criticisms that prevent me from accepting the paper at this stage. They suggest, however, that a revised version might prove acceptable, if you can address their concerns. If you think that you can deal satisfactorily with the criticisms on revision, I would be pleased to see a revised manuscript. We would then return it to the reviewers.

Please ensure that you clearly highlight all changes made in the revised manuscript. Please avoid using 'Tracked changes' in Word files as these are lost in PDF conversion.

I should be grateful if you would also provide a point-by-point response detailing how you have dealt with the points raised by the reviewers in the 'Response to Reviewers' box. Please attend to all of the reviewers' comments. If you do not agree with any of their criticisms or suggestions please explain clearly why this is so.

Reviewer 1

*Advance summary and potential significance to field*

This manuscript investigates dynamic changes in the F-actin cytoskeleton that occur at newly formed boundaries between anterior and posterior compartment cells in the Drosophila abdomen. Compartment boundaries are often associated with elevated levels of F-actin and myosin, and with

© 2023. Published by The Company of Biologists under the terms of the Creative Commons Attribution License (https://creativecommons.org/licenses/by/4.0/).

elevated levels of tension along cell-cell junctions. The authors here took advantage of features of the developing abdomen during metamorphosis to investigate what happens when distinct populations of anterior (A) compartment and posterior (P) compartment cells first contact each other.They report observing a transient increase in F-actin and myosin levels along the new cell-cell junctions at the compartment boundary, and a longer-lasting increase in juctional tension. The experiments appear technically solid, but even if the authors claims are true it seems like a modest advance, and it also seems possible that the authors have over-interpreted or mis-interpreted their results.

*Comments for the author*

The main problem is that the authors assert that the transient increase in F-actin and myosin they characterize is an aspect of compartment boundary formation, when it’s not clear that this is the case.
During pupal development, small clusters of cells that will form the abdominal epidermis of the adult (histoblasts) grow and replace larval epidermal cells (LECs). The histoblasts are initially segregated in distinct A and P compartment nests within each segment, which later contact each other as they replace LECs. However, as the authors note, cell-cell junctions between LECs and histoblasts also have elevated F-actin and myosin levels. Thus it’s possible that the transient elevation of F-actin and myosin along initial A-P junctions is simply inherited from the LEC-histoblast junction that it just replaced. Moreover, as the LECs shrink and die through an apoptotic process, formation of an A-P junction requires formation a new cell-cell junction to replace the junction that was just eliminated by shrinking of the dying cell. It’s possible that the elevated F-actin and myosin occurs as part of this process of rapidly forming a new junction rather than as a consequence of that junction occurring at a compartment boundary. Indeed, loss of a cell through cell death is a bit like a wounding, and wound healing in epithelia is often associated with transient increases in F-actin and myosin that help close the wound. In any of these cases, the transient elevation of F-actin and myosin described would actually have nothing to do with the fact that it occurs at a compartment boundary.

There might be ways that the authors could get around this, eg by using genetic manipulations to switch the compartment identities of histoblast nests (ie comparing what happens at new A-A or P-P cell-cell junctions), or making inducible clones of cells within a histoblast nest that switch compartmental identity.
Or comparing new junctions at A-P boundaries to new junctions formed after eliminating histoblasts in the middle of a nest by laser ablation. As it stands, the authors haven’t convincingly demonstrated what they claim to have shown.

Additional minor point
- The observation that tension is higher even after F-actin and myosin levels are no longer elevated is somewhat surprising. Could the authors check whether myosin activity is elevated by staining for phosphorylated myosin?

Reviewer 2

*Advance summary and potential significance to field*

The work by Wang and Michel et al. ‘Preferential recruitment and stabilization of Myosin II at compartment boundaries in Drosophila‘ studies the mechanical tension and myosin II distributionat cell boundaries between adjacent anterior and posterior histoblast cells in drosophila pupae. Three claims are made: i) tension increases between A and P cells in the first minutes of contact and boundary formation, ii) this increase in tension is accompanied by increased local recruitment of myosin II and iii) myosin II at these sites is stabilised.
The authors show experiments supporting each claim individually. The tension along the boundary lines is measured by following the recoil of adjacent cell-junction vertices after laser ablation, with the finding that the vertex distance increases about three times at AP boundaries compared to AA boundaries. The initial increase and recruitment of myosin II to initial AP boundaries is shown by fluorescence intensity measurements of Sqh-GFP and by following cytoplasmic Sqh:Dendra2 after photoconversion. And FRAP of Sqh-GFP shows an increased immobile fraction at initial AP-boundaries indicating a stabilisation of myosin II. In addition, sequential laser cutting experiments

© 2023. Published by The Company of Biologists under the terms of the Creative Commons Attribution License (https://creativecommons.org/licenses/by/4.0/).

on boundaries of neighbouring cells showed that cortical tension is maintained on a cellular level and is not affected by neighbouring cells at the initial stages of AP boundary formation.
This study provides clear data about the tension between different cell boundaries in the developing fly which is important for the field to understand better the physics of processes acting in developmental processes. However, the question raised in the introduction about the cellular mechanisms by which myosin II accumulates at certain cell boundaries remains unclear, and the authors should consider to be more careful with their conclusions about how much insight about the mechanisms their study reveals. E.g. the observation that myosin II is preferentially recruited onto initial AP boundaries does not reveal how this preferential recruitment is achieved making it a more observational than mechanistic work.
Taking the limitations and concerns outlined below into account, I think the work would after revision be fit for publication in Journal of Cell Sciences.

*Comments for the author*

Major comments:
-Fig. 6 reports about the preferential recruitment of myosin II onto initial AP boundaries and Fig. 5 shows FRAP experiments concluding that myosin II is more immobile or stabilised at initial boundaries. The authors should discuss how these observations can be brought into agreement. If more myosin II from the cytoplasm is recruited to these boundaries, the recovery after photobleaching should be faster or, vice versa, if the myosin II filaments remain longer at the initial boundaries, one would expect that it would be more difficult to recruit additional myosin II filaments there. The time scales of both experiments seem comparable. There could be other reasons for a seemingly higher immobile fraction in FRAP, e.g. a reduced amount of fresh protein to replace the bleached proteins. In the example image shown, the surrounding cells also show decreasing myosin levels. To test the claim of increased stabilisation, could the authors photoconvert sqh:Dendra at the boundary and follow how it gets replaced by non-converted sqh:Dendra?

-The measurements in Fig. 1 show clearly that tension between AP boundaries is elevated compared to AA boundaries at 5 min of boundary formation. However, at which time this tension increases is still not clear. Is there a reason that the tension between PP boundaries was not tested? This would be important to support the suggestion by the authors that it is the previous contact with LEC that leads to the increase in tension.

-the discussion about the two-step model does not address the question how the authors explain the maintenance of high AP boundary tension while the levels of myosin and actin return to levels found in AA boundaries.

Minor comments:
-Line 85, one '(' too much at reference.

-The number of independent pupae used for laser cut experiments should be mentioned.

-Line 156: the title of the paragraph is ambiguous. Simply write, something like 'Mechanical tension is generated autonomously for each cell junction'

-Movie 3 seems not well suited to support the claims. The movie plays very fast and is a bit difficult to follow. It might help if the authors add markers to show LECs which are extruding or to highlight new formations of boundaries.

-Figure 5: Wording in the legend: the whisker of the box plot is not the standard error of the fit, but of the distribution. Could the authors say why they do not show the standard deviation which would better reflect the spread of the data? It would also be good to plot the individual data points like it is done in Fig. 6.

-Figure 6: In A) you should mention that the color bar indicates the fluorescence intensity levels of the following images. Equally, the range of the grey scale should be shown. How is intensity measured along cell boundaries? Via a line scan along the boundary drawn by hand?

© 2023. Published by The Company of Biologists under the terms of the Creative Commons Attribution License (https://creativecommons.org/licenses/by/4.0/). 

-Figure 7: schematic can benefit from some clarification. The arrows for ‘bond’ tension are confusing. What is happening when there is a single arrow on a cell boundary, is there any evidence from the data that cell boundaries shrink in such configurations?

Reviewer 3

*Advance summary and potential significance to field*

Wang and colleagues investigate the mechanisms by which tissue boundaries form.
The authors use the boundaries between anterior-posterior compartments in the abdominal epidermis of Drosophila pupae, which form one junction at a time as cells in between the compartments extrude one-by-one. They find that tension at the boundary (measured by laser ablation) is higher than within compartments, and that increased tension is measurable from the time of initiation of boundary formation. Junctions at the boundary seem to be mechanically buffered, as releasing tension in a junction does not reduce the tension in other junctions along the boundary. As the boundary forms, the authors show that both actin and myosin II transiently accumulate there (for approximately 45-50 min) in a tension-independent manner. Myosin accumulation is associated with its stabilization and preferential recruitment to the AP boundary. The authors conclude that the combination of preferential recruitment and stabilization leads to the transient accumulation of myosin and boundary formation.

*Comments for the author*

This is a nice, well-written paper that I enjoyed reading. The data are clear, and the choice of statistical tests is appropriate. I am a bit concerned about the interpretation of some results and whether they show what the authors are claiming. Thus, I propose to address the following points:

MAJOR
1. Figure 2: The authors argue that tension is generated autonomously at each junction at the boundary based on consecutive ablations. I have two questions about this:
a. What stage along the process of boundary formation (initial discontinuous or continuous) were these experiments conducted? If this was done during the initial or discontinuous stage, then there isn't really a supracellular actomyosin cable that could generate supracellular force, right? And if the cuts were done at the continuous stage, myosin levels have already decreased after the transient increase, so there isn't really a polarized cable either? The authors should clearly indicate when did they do the experiments, and better yet, show the distribution of myosin in the tissues that they cut twice.
b. From the example shown in Figure 2A-D, there are at least two junctions between the two contacts severed. To show that junctions along the boundary are mechanically isolated from each other, the authors should show that ablation of consecutive (immediately adjacent) junctions reproduces the results of Figure 2.
This could be technically challenging, so another possibility is to look at myosin levels (as a proxy for tension), or at another "tension sensor" (eg. vinculin/E-cadherin ratio) in the boundary junctions immediately adjacent to the severed one and determine if there are any changes.

2. Figure 4: the authors argue that myosin localization does not depend on tension based on measurements on severed junctions. However, those junctions are damaged and may be subject to cell repair responses, that often involve myosin accumulation (see work from Susan Parkhurst or Bill Bement's lab). The authors should show that the results are the same if the myosin measurements are done on junctions immediately adjacent to the severed ones along the boundary, which would also more directly address the previous point about the tension (non)autonomy in different junctions at the boundary.

3. Figures 5 and 6: I find the combination of these results confusing. If "cytoplasmic" myosin is preferentially recruited to the AP boundary during the initial phase (Figure 6) ... how is it possible that the FRAP experiments show the largest pool of immobile myosin at that stage (Figure 5)? Wouldn't one expect that if myosin is actively being recruited to the boundary from the cytoplasm, a FRAP experiment would show greater and/or faster recovery than in the situations with no myosin recruitment? Based on Figure 6B, it is hard to tell if the pool of cytoplasmic myosin that is recruited

© 2023. Published by The Company of Biologists under the terms of the Creative Commons Attribution License (https://creativecommons.org/licenses/by/4.0/). 

to the junction is much smaller than the pool that is already there, which could explain a "dominant" impact of junctional myosin on the FRAP data. Are there any differences in the half time of fluorescence recovery in the FRAP experiments (Figure 5H)? Are there differences between the half time of fluorescence recovery in the FRAP assay and the half time of fluorescence recruitment in the photoconversion experiment that could explain these results (e.g. if fluorescence recruitment occurred over longer time scales although based on Figures 5-6 they both seem to occur in the order of t1/2=60 s)?

MINOR
1. While possibly repetitive, the authors should include representative images (or movies) of the data quantified in Figure 1I and Figure 1K (currently only images for Figure 1J are shown).

2. Figure 6: the grayscale signal in the "merge" images is really hard to see. I would suggest using a green-magenta or cyan-red overlay.

TYPOS
1. Line 85: too many parentheses.
2. Line 219: "stabile" should be "stable".

---

**First revision**

Author response to reviewers' comments

We thank the reviewers for their insightful and constructive comments.

**Summary of major changes to the manuscript:**

- We now show that after LEC extrusion Myosin II is only slightly elevated at newly forming junctions between histoblasts located away from the AP boundary. These data show that the elevated levels of Myosin II at the forming AP boundary are not merely a consequence of the prior extrusion of an LEC (**new Supplementary Fig. S3**) but rather a consequence of the forming AP boundary.
- We have now conducted additional laser ablation experiments where we systematically varied the distance between the two consecutively ablated junctions. The obtained data support our previous conclusion that mechanical tension along the AP boundary is generated autonomously for each cell (**new Supplementary Fig. S2**).
- We now show that, as with the partially ablated junction, Myosin levels remain constant also in the junctions immediately adjacent to the partially ablated ones (**revised Figure 4).** These new findings support our previous conclusion that cortical Myosin II localization in histoblasts does not depend on mechanical tension.
- We now discuss how the preferential recruitment of Myosin II towards AP cell junctions does not lead to an overall faster recovery of Myosin II in the FRAP experiments at these junctions: The cortical pool of Myosin II is much larger than the cytoplasmic pool and therefore the cortical pool dominates the kinetics of Sqh-GFP recovery after photobleaching.

**Point-by-point response:**

Reviewer 1 Comments for the Author:
The main problem is that the authors assert that the transient increase in F-actin and myosin they characterize is an aspect of compartment boundary formation, when it's not clear that this is the case. During pupal development, small clusters of cells that will form the abdominal epidermis of the adult (histoblasts) grow and replace larval epidermal cells (LECs). The histoblasts are initially segregated in distinct A and P compartment nests within each segment, which later contact each other as they replace LECs. However, as the authors note, cell-cell junctions between LECs and histoblasts also have elevated F-actin and myosin levels. Thus, it's possible that the transient elevation of F-actin and myosin

© 2023. Published by The Company of Biologists under the terms of the Creative Commons Attribution License (https://creativecommons.org/licenses/by/4.0/).

along initial A-P junctions is simply inherited from the LEC-histoblast junction that it just replaced. Moreover, as the LECs shrink and die through an apoptotic process, formation of an A-P junction requires formation a new cell-cell junction to replace the junction that was just eliminated by shrinking of the dying cell. It's possible that the elevated F-actin and myosin occurs as part of this process of rapidly forming a new junction, rather than as a consequence of that junction occurring at a compartment boundary. Indeed, loss of a cell through cell death is a bit like a wounding, and wound healing in epithelia is often associated with transient increases in F-actin and myosin that help close the wound. In any of these cases, the transient elevation of F-actin and myosin described would actually have nothing to do with the fact that it occurs at a compartment boundary.

There might be ways that the authors could get around this, eg by using genetic manipulations to switch the compartment identities of histoblast nests (ie comparing what happens at new A-A or P-P cell-cell junctions), or making inducible clones of cells within a histoblast nest that switch compartmental identity. Or comparing new junctions at A-P boundaries to new junctions formed after eliminating histoblasts in the middle of a nest by laser ablation. As it stands, the authors haven't convincingly demonstrated what they claim to have shown.
**OUR RESPONSE: We have now used two strategies outlined by the reviewer to test whether the elevated levels of F-actin and Myosin II are merely a consequence of the extrusion of LECs and the subsequent formation of a new junction between histoblasts rather than the consequence of the initial contact of cells during the formation of the AP boundary.**

**First, we have switched compartmental identity by generating clones of histoblasts mutant for *engrailed* and *invected*, genes that together 'select' posterior cell identity. As previously shown, when located in the posterior compartment, clone borders of such clones resemble compartment boundaries (e.g. junctions at these clone borders are under increased mechanical tension (Umetsu et al. (2014) Curr Biol *24*, 1798-1805). Clone borders did not show elevated F-actin levels (see Fig. 1 for Reviewers). This is expected, because the clone border resembles the established compartment boundary. We therefore attempted to find dividing cells in the mutant clone that would form a new junction with the surrounding wild-type cells (i.e. resembling a junction at the newly forming compartment boundary). However, even though we imaged multiple clones over extended periods of time, we could not find dividing cells at the clone border.**

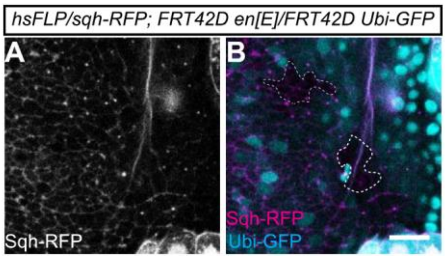


**Figure 1 for reviewers. Image of a time-lapse movie showing the pupal abdominal epidermis carrying *en[E]* mutant clones of cells (mutant for *engrailed* and *invected*) identified by the absence of GFP (blue) and expressing Sqh-RFP (magenta, a marker for Myosin II). Clones located in the posterior compartment are outlined by dashed lines. Scale bar: 15 µm.**

**Second, we analysed Myosin II (Sqh-GFP) levels at newly forming junctions between histoblasts upon LEC extrusion away from the AP boundary. Myosin II levels were only slightly elevated (see new Supplementary Fig. S3). Thus, the elevated levels of Myosin II (and likely also of F-actin) at forming AP junctions are not merely due to LEC extrusion. We revised the manuscript (lines 200-204): "Moreover, Sqh-GFP levels at new cell junctions between histoblasts resulting from the extrusion of an LEC locatedaway from the AP boundary were elevated by 35% compared to existing junctions (Fig. S3). This finding indicates that the elevated Myosin II levels between A and P cells 5 minutes after initial contact were notto a large extent a consequence of the LEC extrusion."**

© 2023. Published by The Company of Biologists under the terms of the Creative Commons Attribution License (https://creativecommons.org/licenses/by/4.0/). 

Additional minor point

- The observation that tension is higher even after F-actin and myosin levels are no longer elevated is somewhat surprising. Could the authors check whether myosin activity is elevated by staining for phosphorylated myosin?

**OUR RESPONSE: We have now stained the tissue with an antibody specific for phosphorylated-Myosin II. Phospho-Myosin II was not elevated along the AP boundary. We revised the manuscript (lines 204-206): "Finally, immunostaining of the pupal abdominal epidermis using an anti-phospho Myosin antibody, which reveals active Myosin (Craig et al., 1983; Jordan and Karess, 1997), did not show elevated levels at junctions along the established AP boundary (Fig. S4)."**

Reviewer 2 Comments for the Author:

Major comments:
-Fig. 6 reports about the preferential recruitment of myosin II onto initial AP boundaries and Fig. 5 shows FRAP experiments concluding that myosin II is more immobile or stabilised at initial boundaries. The authors should discuss how these observations can be brought into agreement. If more myosin II from the cytoplasm is recruited to these boundaries, the recovery after photobleaching should be faster or, vice versa, if the myosin II filaments remain longer at the initial boundaries, one would expect that it would be more difficult to recruit additional myosin II filaments there. The time scales of both experiments seem comparable. There could be other reasons for a seemingly higher immobile fraction in FRAP, e.g. a reduced amount of fresh protein to replace the bleached proteins. In the example image shown, the surrounding cells also show decreasing myosin levels. To test the claim of increased stabilisation, could the authors photoconvert sqh:Dendra at the boundary and follow how it gets replaced by non-converted sqh:Dendra?

**OUR RESPONSE: We have now discussed these observations (line 389-394): "It might have been expected that the preferential recruitment of Myosin II towards AP cell junctions may also lead to a faster recovery of Sqh-GFP at these junctions after photobleaching; however, this was not observed (Fig. 5H). We therefore speculate that the cortical pool of Myosin II is much larger than the cytoplasmic pool and that the cortical pool therefore dominates the kinetics of Sqh-GFP recovery after photobleaching." We also like to point out that the time scales of the recovery after photobleaching and the photoconvesion experiments are very different. For the FRAP experiment, the half-time of recovery is about 10-20 secs (Fig. 5H). However, the recruitment of photoconverted Myosin to the cell junction is much faster. At the first time point after photoconversion (i.e. 0 sec) the steady-state of photoconverted Myosin recruitment at the cell cortex is already reached (Fig. 6B-D). Finally, we note that for technical reasons we are unable to locally photoconvert sqh-Dendra at cell boundaries without photoconverting sqh-Dendra in the surrounding cytoplasm. Apparently, the laser light used for photoconversion cannot be sufficiently focussed to a small region of interest.**

-The measurements in Fig. 1 show clearly that tension between AP boundaries is elevated compared to AA boundaries at 5 min of boundary formation. However, at which time this tension increases is still not clear. Is there a reason that the tension between PP boundaries was not tested? This would be important to support the suggestion by the authors that it is the previous contact with LEC that leads to the increase in tension.

**OUR RESPONSE: We had previously shown that mechanical tension is similar at AA and PP cell junctions in the histoblasts (Umetsu et al. (2014) Curr Biol *24,* 1798-1805) and also in wing discs (Landsberg et al. (2009) Curr Biol *19,* 1950-1955). We therefore limited our analysis this time to the AA and AP cell junctions.**

-the discussion about the two-step model does not address the question how the authors explain the maintenance of high AP boundary tension while the levels of myosin and actin return to levels found in AA boundaries.

© 2023. Published by The Company of Biologists under the terms of the Creative Commons Attribution License (https://creativecommons.org/licenses/by/4.0/).

**OUR RESPONSE: We discuss the question how high AP boundary tension can be maintained while the levels of Myosin II and F-actin return to levels found at AA cell junctions (line 345): "How mechanical tension on cell junctions remains increased is not known, but may depend on the structure of the cortical actomyosin network, which determine the efficiency by which Myosin II molecules exert forces on cell junctions (Munjal and Lecuit, 2014)."**

Minor comments:
-Line 85, one '(' too much at reference.

**OUR RESPONSE: Thank you, we corrected this error.**

-The number of independent pupae used for laser cut experiments should be mentioned.

**OUR RESPONSE: We now mention the number of independent pupae used for the laser cut experiments.**

-Line 156: the title of the paragraph is ambiguous. Simply write, something like 'Mechanical tension is generated autonomously for each cell junction'

**OUR RESPONSE: We revised the title as suggested by the reviewer.**

-Movie 3 seems not well suited to support the claims. The movie plays very fast and is a bit difficult to follow. It might help if the authors add markers to show LECs which are extruding or to highlight new formations of boundaries.
**OUR RESPONSE: We have now slowed down the movie and have added markers to indicate the extruding LECs.**

-Figure 5: Wording in the legend: the whisker of the box plot is not the standard error of the fit, but of the distribution. Could the authors say why they do not show the standard deviation which would better reflect the spread of the data? It would also be good to plot the individual data points like it is done in Fig. 6.

**OUR RESPONSE: We estimated the immobile fraction of Sqh-GFP by fitting the recovery curves to a double exponential function (see Materials and Methods, lines 533-540). The error was then computed from the standard error of the fit parameters (see lines 541-542).**

-Figure 6: In A) you should mention that the color bar indicates the fluorescence intensity levels of the following images. Equally, the range of the grey scale should be shown. How is intensity measured along cell boundaries? Via a line scan along the boundary drawn by hand?

**OUR RESPONSE: We now mention that the color bar indicates fluorescence intensities of photoconverted Sqh::Dendra2 in (B,C,D). We mention in the Materials and Methods that the intensities along cell boundaries were manually drawn (line 564).**

-Figure 7: schematic can benefit from some clarification. The arrows for 'bond' tension are confusing. What is happening when there is a single arrow on a cell boundary, is there any evidence from the data that cell boundaries shrink in such configurations?

**OUR RESPONSE: We apologize for the confusion. We revised the schematic to always have two arrows pointing towards each other per junction.**

Reviewer 3 Comments for the Author:
This is a nice, well-written paper that I enjoyed reading. The data are clear, and the choice of statistical tests is appropriate. I am a bit concerned about the interpretation of some results and whether they show what the authors are claiming. Thus, I propose to address the following points:

© 2023. Published by The Company of Biologists under the terms of the Creative Commons Attribution License (https://creativecommons.org/licenses/by/4.0/).

MAJOR

1. Figure 2: The authors argue that tension is generated autonomously at each junction at the boundary based on consecutive ablations. I have two questions about this:

a. What stage along the process of boundary formation (initial, discontinuous or continuous) were these experiments conducted? If this was done during the initial or discontinuous stage, then there isn't really a supracellular actomyosin cable that could generate supracellular force, right? And if the cuts were done at the continuous stage, myosin levels have already decreased after the transient increase, so there isn't really a polarized cable either? The authors should clearly indicate when did they do the experiments, and better yet, show the distribution of myosin in the tissues that they cut twice.

**OUR RESPONSE: The experiments were conducted at the continuous stage. As the reviewer points out, at the initial or discontinuous stages there are only few neighboring junctions along the AP boundary which makes it difficult to perform consecutive laser ablations. We now revised the manuscript (lines166- 168): "…we used focused laser light to ablate consecutively two cell junctions along the established AP boundary ..." Please note that we do not use the term 'continuous stage', because we introduce the three stages only later in the manuscript.**

b. From the example shown in Figure 2A-D, there are at least two junctions between the two contacts severed. To show that junctions along the boundary are mechanically isolated from each other, the authors should show that ablation of consecutive (immediately adjacent) junctions reproduces the results of Figure 2. This could be technically challenging, so another possibility is to look at myosin levels (as a proxy for tension), or at another "tension sensor" (eg. vinculin/E-cadherin ratio) in the boundary junctions immediately adjacent to the severed one and determine if there are any changes.

**OUR RESPONSE: The motivation for this experiment was to test for collective effects across adjacent cells, as would for example be generated by a supracellular cable. Two immediately adjacent junctions (except in rare cases of 4-way junctions), however, come to lay in the same cell (see new Supplemental Fig. S2A). Thus, ablation of immediately adjacent junctions measures to a large extent collective effects within a cell. By including one or more junctions in between the two ablated cell junctions, we ablated cell junctions of different (adjacent) cells. We and Scarpa et al. previously used a similar strategy, leaving one or more cell junctions in between the ablated junctions to ablate junctions of neighboring cells (Rudolf et al. (2015) Development *142*, 3845-3858; Scarpa et al., (2018) Dev Cell *47*, 727-740).**

**We have now systematically conducted laser ablation experiments where we ablated immediately adjacent junctions, ablated two junctions with one intervening or with two or more intervening junctions. We find for ablating immediately adjacent junctions that the initial velocity of vertex displacement is significantly lower in the second ablation compared to the first ablation (new Supplemental Fig. S2).**
**However, in the cases of one intervening or two or more intervening junctions, the initial velocities of vertex displacement are similar between the first and second ablations (new Supplemental Fig. S2), as we originally showed in Fig. 2. We conclude that collective effects contribute to mechanical tension on cell junctions within the same cell, but not to mechanical tension across neighboring cells. We revised the manuscript as follows (lines 169-179): "We either ablated two immediately neighboring junctions of the same cell (case I) or two nearby junctions in neighboring cells (case II) along the AP boundary. In both cases, we ablated the two junctions with a time delay and recorded the resulting displacement of the two vertices at the ends of the ablated cell junctions by time-lapse microscopy. For case I, the initial velocity of vertex displacement was significantly decreased upon ablation of the second cell junction compared to the first cell junction (Fig. S2). However, for case II, the initial velocity of vertex displacement was indistinguishable upon ablation of the first and second cell junction (Fig. 2B-F, Fig. S2, Movie 2). Thus, while collective effects may contribute to the mechanical tension of cell junctions within a cell, mechanical tension along the AP boundary is generated autonomously for each cell."**

© 2023. Published by The Company of Biologists under the terms of the Creative Commons Attribution License (https://creativecommons.org/licenses/by/4.0/).

2. Figure 4: the authors argue that myosin localization does not depend on tension based on measurements on severed junctions. However, those junctions are damaged, and may be subject to cell repair responses, that often involve myosin accumulation (see work from Susan Parkhurst or Bill Bement's lab). The authors should show that the results are the same if the myosin measurements are done on junctions immediately adjacent to the severed ones along the boundary, which would also more directly address the previous point about the tension (non)autonomy in different junctions at the boundary.

**OUR RESPONSE: We have now analysed Myosin levels in junctions immediately adjacent to the partially ablated ones. We show that, as with the partially ablated junction, Myosin levels remaining fairly constant also in the junctions immediately adjacent to the partially ablated ones (revised Figure 4). We revised the manuscript (lines 216-222): "We then monitored the level of Myosin II (Sqh-GFP) on the remaining part of the junction or on an immmediately neighboring junction by time-lapse microscopy. If cortical localization of Myosin II depends on mechanical tension, we expected that Myosin II levels would decline upon ablation of a junction. At control (unablated) cell junctions, Myosin II levels remained fairly constant over time (Fig. 4A-C, Movie 4). Similarly, upon ablation, Myosin II levels remained fairly constant both on the remaining part of the ablated junction and on immediately neighboring junctions (Fig. 4C-E, Movie 4)."**

3. Figures 5 and 6: I find the combination of these results confusing. If "cytoplasmic" myosin is preferentially recruited to the AP boundary during the initial phase (Figure 6) ... how is it possible that the FRAP experiments show the largest pool of immobile myosin at that stage (Figure 5)? Wouldn't one expect that if myosin is actively being recruited to the boundary from the cytoplasm, a FRAP experiment would show greater and/or faster recovery than in the situations with no myosin recruitment? Based on Figure 6B, it is hard to tell if the pool of cytoplasmic myosin that is recruited to the junction is much smaller than the pool that is already there, which could explain a "dominant" impact of junctional myosin on the FRAP data. Are there any differences in the half time of fluorescence recovery in the FRAP experiments (Figure 5H)? Are there differences between the half time of fluorescence recovery in the FRAP assay and the half time of fluorescence recruitment in the photoconversion experiment that could explain these results (e.g. if fluorescence recruitment occurred over longer time scales, although based on Figures 5-6 they both seem to occur in the order of t1/2=60 s)?

**OUR RESPONSE: We have now discussed these observations (line 390-395): "It might have been expected that the preferential recruitment of Myosin II towards AP cell junctions may also lead to a faster recovery of Sqh-GFP at these junctions after photobleaching; however, this was not observed (Fig. 5H). We therefore speculate that the cortical pool of Myosin II is much larger than the cytoplasmic pool and that the cortical pool therefore dominates the kinetics of Sqh-GFP recovery after photobleaching." We also like to point out that the time scales of the recovery after photobleaching and the photoconvesion experiments are very different. For the FRAP experiment, the half-time of recovery is about 10-20 secs (Fig. 5H). However, the recruitment of photoconverted Myosin to the cell junction is much faster. At the first time point after photoconversion (i.e. 0 sec) the steady-state of photoconverted Myosin recruitment at the cell cortex is already reached (Fig. 6B-D).**

MINOR
1. While possibly repetitive, the authors should include representative images (or movies) of the data quantified in Figure 1I and Figure 1K (currently only images for Figure 1J are shown).

**OUR RESPONSE: We now show representative images of the data quantified in Figure 1I and 1K in the new Supplementary Fig. S1.**

2. Figure 6: the grayscale signal in the "merge" images is really hard to see. I would suggest using a green-magenta or cyan-red overlay.

**OUR RESPONSE: We have made green-magenta and cyan-red overlays. However, in these overlays the pixel intensity distribution of the converted Sqh::Dendra2 is much harder to see compared to the original overlay. We therefore prefer to keep the original overlay.**

© 2023. Published by The Company of Biologists under the terms of the Creative Commons Attribution License (https://creativecommons.org/licenses/by/4.0/).

TYPOS
1. Line 85: too many parentheses.
2. Line 219: "stabile" should be "stable".

**OUR RESPONSE: Thanks, we corrected the typos.**

---

Second decision letter

MS ID#: JOCES/2022/260447

MS TITLE: Preferential recruitment and stabilization of Myosin II at compartment boundaries in Drosophila

AUTHORS: Jing Wang, Marcus Michel, Lisa Bialas, Giulia Pierini, and Christian Dahmann
ARTICLE TYPE: Research Article

I am happy to tell you that your manuscript has been accepted for publication in Journal of Cell Science, pending standard ethics checks. If you wish to make a small change to your manuscript to clarify the point review 3 raises, you should be able to do so during the production process.

Reviewer 1

*Advance summary and potential significance to field*

This manuscript investigates dynamic changes in the F-actin cytoskeleton that occur at newly formed boundaries between anterior and posterior compartment cells in the Drosophila abdomen. Compartment boundaries are often associated with elevated levels of F-actin and myosin, and with elevated levels of tension along cell-cell junctions. The authors here took advantage of features of the developing abdomen during metamorphosis to investigate what happens when distinct populations of anterior (A) compartment and posterior (P) compartment cells first contact each other.They report observing a transient increase in F-actin and myosin levels along the new cell-cell junctions at the compartment boundary, and a longer-lasting increase in juctional tension.

*Comments for the author*

In this revised manuscript, the authors have satisfactorily addressed the concerns raised in the initial review and we do not have any additional issues that need to be addressed.

Reviewer 2

*Advance summary and potential significance to field*

The authors have done a fantastic job in addressing all points raised by the reviewers.

*Comments for the author*

all points were addressed satisfactorily. I have no further comments.

Reviewer 3

*Advance summary and potential significance to field*

Wang and colleagues investigate the mechanisms by which tissue boundaries form.

© 2023. Published by The Company of Biologists under the terms of the Creative Commons Attribution License (https://creativecommons.org/licenses/by/4.0/).

*Comments for the author*

The authors have addressed most of my concerns. There is just one point that is still not clear to me. They argue that cutting adjacent junctions means cutting junctions belonging to one cell ... which is true on one side of the boundary, but not on the side (Figure S2). I would say that it is hard to distinguish if the reduced velocity of the second cut in that scenario is due to the immediate connection between the two junctions across cells or within a cell. In fact, if as the authors argue the reduced velocity is caused by the two cuts being done to a single cell, then one would expect that cutting a junction perpendicular to the boundary followed by a boundary junction, with both junctions connected and belonging to the same cell, would lead to a lower initial velocity for the second (boundary) cut as compared to the first (perpendicular) cut, or compared to "first cuts" directly at the boundary.

© 2023. Published by The Company of Biologists under the terms of the Creative Commons Attribution License (https://creativecommons.org/licenses/by/4.0/).
